# Multi-omics analysis of pediatric minimally differentiated acute myeloid leukemia reveals *RUNX1*-driven stemness and chemoresistance

**DOI:** 10.1038/s41375-026-02967-6

**Published:** 2026-04-29

**Authors:** Tatsuya Kamitori, Satoshi Saida, Kazuki Mitani, Shinichi Tsujimoto, Hiroaki Goto, Hirofumi Shibata, Ryo Akazawa, Kiyotaka Isobe, Hiroo Ueno, Nobuyuki Kakiuchi, Akiko M. Saito, Mitsuteru Hiwatari, Ko Kudo, Shinsuke Hirabayashi, Kohei Fukuoka, Katsuyoshi Koh, Takashi Taga, Hirohito Kubota, Itaru Kato, Katsutsugu Umeda, Souichi Adachi, Tomoko Kawai, Daisuke Tomizawa, Junji Ikeda, Norio Shiba, Yasuhide Hayashi, Seishi Ogawa, Junko Takita

**Affiliations:** 1 Department of Pediatrics, Graduate School of Medicine, Kyoto University, Kyoto, Japan; 2 Department of Hematology/Oncology, Saitama Children’s Medical Center, Saitama, Japan; 3 Department of Pediatrics, Graduate School of Medicine, Yokohama City University, Yokohama, Japan; 4 Division of Hematology/Oncology, Kanagawa Children’s Medical Center, Yokohama, Japan; 5 Department of Pediatric Oncology, National Cancer Center Hospital, Tokyo, Japan; 6 Department of Pathology and Tumor Biology, Graduate School of Medicine, Kyoto University, Kyoto, Japan; 7 The Hakubi Center for Advanced Research, Kyoto University, Kyoto, Japan; 8 Clinical Research Center, NHO Nagoya Medical Center, Nagoya, Japan; 9 Department of Pediatrics, Teikyo University School of Medicine, Tokyo, Japan; 10 Department of Pediatrics, Hirosaki University Graduate School of Medicine, Hirosaki, Japan; 11 Department of Pediatrics, Hokkaido University Hospital, Sapporo, Japan; 12 Department of Pediatrics, Shiga University of Medical Science, Otsu, Japan; 13 Human Health Science, Graduate School of Medicine, Kyoto University, Kyoto, Japan; 14 Department of Maternal Fetal Biology, National Center for Child Health and Development, Tokyo, Japan; 15 Division of Leukemia and Lymphoma, Children’s Cancer Center, National Center for Child Health and Development, Tokyo, Japan; 16 Gunma Children’s Medical Center, Shibukawa, Japan; 17 Institute for the Advanced Study of Human Biology, Kyoto University, Kyoto, Japan; 18 Department of Innovative Medicine, Faculty of Medicine, Kindai University, Osaka, Japan

**Keywords:** Acute myeloid leukaemia, Cancer genomics

## Abstract

Minimally differentiated acute myeloid leukemia (AML-M0) is a rare and therapeutically challenging subgroup of AML characterized by immature hematopoietic stem cell-like features. To uncover the molecular basis, we conducted a comprehensive multi-omics analysis of 23 pediatric AML-M0 cases and compared them with 1483 leukemia samples. AML-M0 formed a characteristic group that exhibited global DNA hypermethylation and transcriptional suppression, particularly downregulation of genes related to oxidative phosphorylation and ribosome assembly compared to non-M0 AML. Genomic profiling revealed frequent loss-of-function alterations in *RUNX1* (26%) and *ETV6* (22%), along with activating mutations in signaling pathways (83%), such as *RAS*, *FLT3*, and *JAK*. Notably, *RUNX1* alterations were significantly associated with a poor prognosis. Functional analyses using a CRISPR/Cas9-mediated *RUNX1* knockout in a pediatric AML-M0 cell line showed stem cell-like transcriptional features and reduced expression of genes related to oxidative phosphorylation and ribosomal pathways. *RUNX1* disruption was also associated with reduced in vitro sensitivity to multiple drugs, including cytarabine and anthracyclines. Our study provides the most comprehensive molecular characterization of pediatric AML-M0 to date and identifies *RUNX1* alterations as important biological and clinical determinants. These insights highlight the potential strategies for precision therapy, including hypomethylating agents, signaling inhibitors, and metabolic targeting, to improve outcomes.

## Introduction

Minimally differentiated acute myeloid leukemia (AML-M0) is a unique subgroup of AML characterized by a lack of morphological myeloid differentiation and accounts for <5% of AML cases [1–3]. In the French–American–British (FAB) classification, AML-M0 is designated as the “M0” subtype [2], whereas the World Health Organization (WHO) classified it as “Acute myeloid leukemia with minimal differentiation” in the absence of defining genetic abnormalities [3]. AML-M0 is associated with a poor prognosis in both pediatric and adult cohorts [4, 5], and some treatment protocols classify it as high-risk [6, 7]. However, the mechanisms underlying treatment resistance are poorly understood.

Previous studies on AML-M0 have primarily focused on adult cases, identifying frequent *RUNX1* mutations (30–40%) as well as *ETV6* abnormalities and mutations in the RAS signaling pathway, suggesting their involvement in the pathogenesis of this disease [8–10]. Similar abnormalities have been reported in pediatric AML-M0 and M1 cases [11]. However, comprehensive data on the genomic, transcriptomic, and epigenetic profiles of AML-M0 remain limited, particularly for pediatric patients. Given the well-documented differences between pediatric and adult AML [11], a detailed investigation of pediatric AML-M0 is essential for refining the diagnosis, risk stratification, and therapeutic approaches.

AML-M0 is a subgroup of AML that shares biological features and exists along a continuous spectrum with immature phenotype leukemias, such as acute leukemia of ambiguous lineage (ALAL) and early T-cell precursor acute lymphoblastic leukemia (ETP-ALL) [12]. According to WHO criteria, AML with minimal differentiation is immunophenotypically defined by the expression of at least two myeloid markers (e.g., CD13, CD33, and CD117) in the absence of lymphoid markers [3]. However, distinguishing AML-M0 from immature phenotype leukemia can be clinically challenging owing to their overlapping features. Recent next-generation sequencing-based analyses of ALAL have revealed both subtype-specific and shared genomic events [13]. Similarly, a comprehensive characterization of AML-M0 is essential to clarify its relationship with other leukemias and improve our understanding of its underlying biology.

In this study, we performed a comprehensive multi-omics analysis to characterize the genomic, transcriptomic, and epigenetic features of AML-M0 and elucidate its relationship with other acute leukemias. We aimed to establish an improved classification framework and optimized treatment strategies.

## Materials and methods

### Patients and materials

A total of 23 pediatric AML-M0 cases were included in this study. The diagnosis was strictly confirmed through a central review of the Japan Children’s Cancer Group (JCCG) and evaluation of flow cytometry data. Morphologically, all patients met the criteria for “M0” in FAB classification. According to the diagnostic criteria of “AML with minimal differentiation” in the WHO classification [3], blast cells must express at least two myeloid-associated markers (CD13, CD117, and/or CD33) and be negative for lymphoid markers. Based on these criteria, seven of 23 AML-M0 cases were finally classified in the “AML with minimal differentiation” category, and sixteen were in the “AML with defining genetic abnormalities” category under the WHO classification. Before sample collection, written informed consent was obtained from patients or their guardians. This study was conducted in accordance with the Declaration of Helsinki and approved by the ethics committees of the JCCG and Kyoto University. Clinical and sample information, in addition to sequencing details, is provided in Supplementary Table S1. A detailed analysis of case 9 was reported previously [14].

Additionally, we analyzed sequencing data from pediatric acute leukemia and adult AML cohorts [13, 15–24]. Notably, the FAB classifications of all cases included in the AML-12 and AML-05 clinical trials of the Japanese Pediatric Leukemia and Lymphoma Study Group (JPLSG, now part of the subcommittee on hematological malignancies of the JCCG) were strictly based on a central review by the JCCG, and data from these cohorts were used in a comparative analysis with non-M0 AML.

### DNA sequencing

Tumor and germline DNA were extracted from the bone marrow or peripheral blood mononuclear cells of the patients using a QIAamp DNA Mini Kit (QIAGEN) according to the manufacturer’s instructions. Whole-exome sequencing (WES), low-depth whole-genome sequencing (WGS), and high-depth WGS were performed in 23, 15, and 4 cases, respectively.

The sequencing reads were aligned to the human reference genome (GRCh37/hg19) using the Burrows-Wheeler Aligner (v.0.7.8) with default parameter settings. Single-nucleotide variants (SNVs) and short insertions/deletions (indels) were called using the Genomon pipeline version 2.6 (https://github.com/Genomon-Project).

### Detection of copy number alterations and structural variations

Copy number alterations (CNAs) were analyzed using sequencing data, with WES and Target-seq processed through our in-house pipeline, CNACS (https://github.com/papaemmelab/toil_cnacs), and WGS analyzed using the CNVkit [25]. Structural variations were detected using the Genomon pipeline (v.2.6).

### RNA sequencing

Total RNA was extracted using QIAzol Lysis Reagent and the miRNeasy Mini Kit (QIAGEN) following the manufacturer’s protocol. Sequencing reads were aligned to GRCh37/hg19 and counted for each gene using the Genomon pipeline (version 2.6). Fusion transcripts were detected using the Genomon pipeline (version 2.6), and previously reported leukemia-associated fusions were extracted.

### Gene expression analysis

Gene expression levels were normalized, transformed using the R package DESeq2 [26], and subjected to subsequent analyses. Differential expression analysis was performed using the DESeq2 package. Gene set enrichment analysis (GSEA) was performed using GSEA software [27] (v4.3.2) and the Molecular Signatures Database [28, 29] (v2023.2). A pre-ranked GSEA was performed using Metascape software [30] (v3.5.20240901). Single-sample level gene set enrichment was calculated using the R package GSVA [31] with default parameters. Multiple subtypes of pediatric leukemia were analyzed in both the Japanese leukemia and TARGET cohorts, including JPLSG AML-12 (*N* = 254) [15], JPLSG AML-05 (*N* = 139) [16], and TARGET-AML (*N* = 271) [20], as well as Japanese T-ALL (*N* = 123) [19] and B-ALL (*N* = 116) [18] cohorts and TARGET-ALL cohorts (*N* = 86 for ALAL [13], *N* = 265 for T-ALL [22], and *N* = 137 for B-ALL [21]. A comparative analysis of pediatric AML-M0 and non-M0 AML was performed using only Japanese AML cohorts, as their FAB subtypes were confirmed by a central review of the JCCG/JPLSG.

### DNA methylation analysis

Genomic DNA (250 ng) was bisulfite-converted using an EZ DNA Methylation Kit (Zymo Research). The converted DNA was then amplified, enzymatically fragmented, purified, and hybridized to the Infinium MethylationEPIC v1.0 BeadChip (Illumina), according to the manufacturer’s protocols. Imaging was performed using an iScan system (Illumina). Data filtering, imputation, and normalization were conducted, and the β-values were calculated using the R package ChAMP [32] (v2.20.1) with default parameters. Differentially methylated probes (DMPs) were identified using the champ.DMP function in the ChAMP package. Probe annotations for the EPIC arrays were sourced from the Infinium MethylationEPIC v1.0 B5 Manifest File (https://jp.support.illumina.com/downloads/infinium-methylationepic-v1-0-product-files.html).

### Dual-omics integrative clustering

For integrative unsupervised clustering, the top 1000 variably expressed genes and the top 1000 most variably methylated probes were selected based on their median absolute deviations. The rlog and M-value matrices were z-score-standardized, and the Euclidean distances between the samples were calculated. These distance matrices were converted into affinity matrices and fused using the similarity network fusion algorithm implemented in the R package SNFtool [33]. The resulting integrated matrix was subjected to consensus clustering using the R package ConsensusClusterPlus [34].

### Cells and cell culture

YCU-AML2 [35] and derivative cell lines were cultured at a humidified incubator (37 °C/5% CO_2_) in RPMI 1640 medium supplemented with 10% fetal calf serum (FCS) and 1% penicillin/streptomycin.

### *RUNX1* knock out using CRISPR-Cas9

*RUNX1*-knockout cells were generated through sequential transduction of Cas9 and guide RNA (gRNA) targeting *RUNX1*. Stable expression of Cas9 in YCU-AML2 cells (YCU-AML2-Cas9) was achieved by transduction with a lentiviral vector, pKLV2-EF1a-Cas9GFP-W (Addgene plasmid #200100) in the presence of 5 µg/mL polybrene. Subsequently, the GFP-positive cells were sorted using an SH800 cell sorter (SONY). Two sgRNA sequences targeting exon 5 of *RUNX1* (Supplementary Table S2) were cloned into pKLV-U6gRNA(Bbsl)-PGKpuro2ABFP (Addgene plasmid #50946) and co-transduced into the YCU-AML2-Cas9 cells with 5 µg/mL of polybrene. Mock control cells were generated using the original #50946 vector. Following transduction, the cells were subjected to puromycin selection (1 µg/mL) for 7 days. Cells expressing high GFP were sorted to enrich the *RUNX1* knocked-out population.

### Cell proliferation assay

Cell proliferation was analyzed using Cell Counting Kit-8 (Dojindo Laboratories) according to the manufacturer’s instructions. The sample absorbance was measured at 450 nm using an iMark Microplate Reader (Bio-Rad). Absorbance values were normalized by dividing them by the absorbance on day 1, and the relative values were used to evaluate cell proliferation.

### Cell cycle assay

Cell cycle assays were performed using a Cell Cycle Assay Kit (Red) (Tokyo Chemical Industry Co., Ltd.) according to the manufacturer’s protocol.

### Colony formation assay

Colony formation assays were performed using YCU-AML2 cells and MethoCult H4434 Classic (STEMCELL Technologies) according to the manufacturer’s instructions.

### Seahorse extracellular flux assay

The real-time ATP assay was performed using a Seahorse XF Real-Time ATP Rate Assay Kit (Agilent Technologies, 103592-100) according to the manufacturer’s instructions on a Seahorse XFe96 Analyzer. YCU-AML2 cells were suspended in XF RPMI medium (pH 7.4; Agilent Technologies, 103576-100) supplemented with 10 mM glucose (Agilent Technologies, 103577-100), 1 mM pyruvate (Agilent Technologies, 103578-100) and 2 mM L-glutamine (Agilent Technologies, 103579-100). 1.5 × 10e5 cells were added into each well of Seahorse XFe96/XF Pro Cell Culture Microplates (Agilent Technologies, 103794-100) coated with Cell-Tak Cell and Tissue Adhesive (Corning, 354240) and incubated for one hour at 37 °C in a non-CO2 incubator prior to the assay. Oxygen consumption rate (OCR) and extracellular acidification rate (ECAR) were measured at baseline and following the sequential injection of oligomycin (1.5 µM) and rotenone/antimycin A (0.5 µM) at the indicated time points.

### High-throughput drug sensitivity screening

High-throughput drug sensitivity screening was performed as described in the previous report [36]. Briefly, cells were seeded at 1 × 10^4^ viable cells/10 µL onto a 384-well plate that was preloaded with 10 µl of culture medium containing 1 of 60 drugs at 4 serially diluted concentrations (× 1, × 1/5, × 1/25, × 1/125). The drugs and their final concentrations are listed in Supplementary Table S3. After a 4-day incubation period, cell viability was assessed using the CellTiter-Glo luminescent assay with a GloMax plate reader (Promega). To compare drug sensitivities between samples the drug effect score (DES) was utilized as follows: DES = [(100− % survival at 1/125 dilution) * ln(125) + (100− % survival at 1/25 dilution) * ln(25) + (100− % survival at 1/5 dilution) * ln(5) + (100− % survival at no dilution)] / [ln(125) + ln(25) + ln(5) + 1]. A DES of 100 indicated that the vector killed all cells at every tested concentration, whereas a DES of 0 indicated that the drug had no effect.

### Statistical analysis

Statistical analyses were performed using GraphPad Prism software (version 9.5.1). Unless otherwise specified, the association between categorical variables was tested using Fisher’s exact test. The Mann–Whitney U test was used to compare quantitative variables. All statistical tests were two-sided. In the survival analysis, the survival curve was estimated using the Kaplan–Meier method, and the log-rank test was used to compare outcomes. Multivariate analysis was performed using the Cox regression analysis.

## Results

### Comprehensive genetic analysis reveals distinct molecular features in pediatric AML-M0

We included 23 pediatric AML-M0 patients who were strictly diagnosed through a central review by the JCCG and evaluation of flow cytometry data. Most early studies on AML-M0 were based on “classic” criteria, defined as MPO negativity, absence of B- and T-lineage markers, and positivity for CD13 and/or CD33 [1, 2]. However, these criteria include acute undifferentiated leukemia (AUL) and other immature leukemia subtypes based on the current criteria. In this study, the blast cells expressed at least two myeloid-associated markers (CD13, CD117, and/or CD33). The clinical characteristics of the patients are summarized in Supplementary Table S1. To comprehensively characterize the genomic landscape of pediatric AML-M0, we performed WES in 23 patients and WGS in 19 patients. We compared our results with those from pediatric patients with non-M0 AML and other types of leukemia in publicly available databases.

**Fig. 1:**
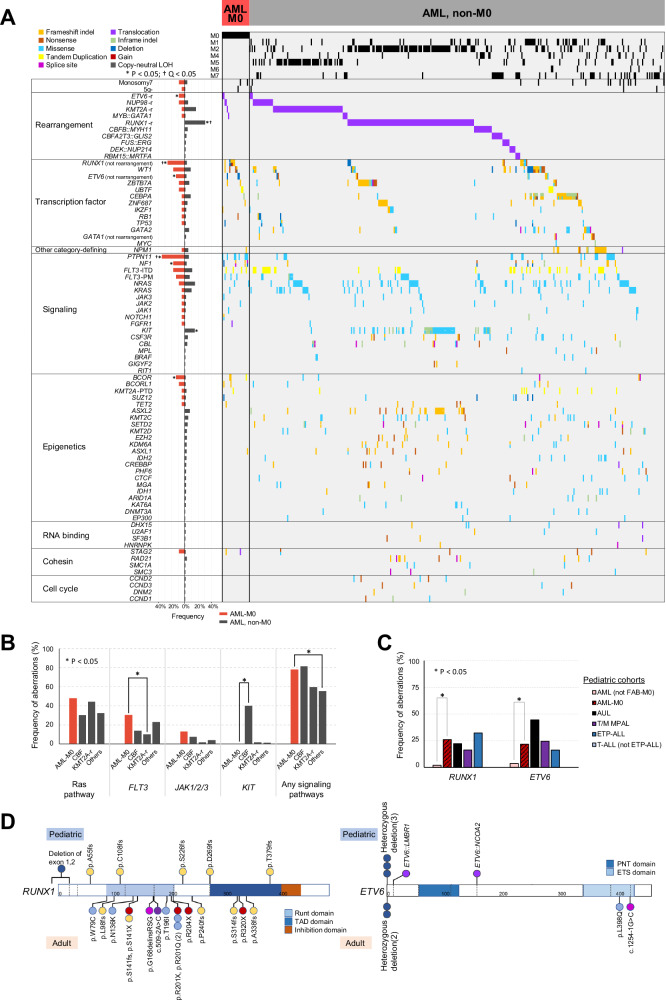
Landscape of genomic alterations in pediatric AML-M0. **A** Cytogenetic and genomic alterations in pediatric AML-M0 (*n* = 23) and non-M0 pediatric AML patients (*n* = 351). Each column represents a single scenario. Alteration frequencies are shown on the left-hand side. **B** Comparative frequencies of signaling pathway mutations between AML-M0 and various subtypes of pediatric AML, including those with core-binding factor (CBF) rearrangements, such as *RUNX1::RUNX1T1* and *CBFB::MYH11*, *KMT2A* rearrangements, and other non-M0 pediatric AML cases. **C** The frequencies of *RUNX1* loss-of-function alterations (left) and *ETV6* aberrations (right) observed in pediatric leukemia subtypes (non-M0 AML, *n* = 351; AML-M0, *n* = 23; AUL, *n* = 9; T/myeloid MPAL, *n* = 49; ETP-ALL, *n* = 31; and non-ETP T-ALL, *n* = 90). **D** Details of the alterations in *RUNX1* (left) and *ETV6* (right) observed in pediatric (upper) and adult (lower) AML-M0 cohorts.

The most frequent genetic alterations in AML-M0 were *RUNX1* (26%) and *ETV6* (22%). After correction for multiple testing, *RUNX1* and *PTPN11* were found to be significantly more prevalent than in non-M0 pediatric AML (Fig. 1A and Supplementary Tables S4–7). Other recurrent alterations, including *ETV6*, *NF1*, and *BCOR*, showed a tendency toward a higher prevalence in AML-M0, but these differences were not statistically significant after correction for multiple comparisons. Most *RUNX1* and *ETV6* aberrations are frameshift, nonsense, or heterozygous deletions that are likely to result in loss-of-function alterations.

Category-defining genomic lesions, as recently described in pediatric AML [11], including *UBTF* tandem duplications (TD), *CEBPA* bZIP domain mutations, *NPM1* mutations, *NUP98::NSD1* fusion, and *MYB::GATA1* fusion, have also been identified in individual cases. Notably, activating mutations in signaling pathways were identified in 83% of the cases, with mutations in RAS pathway genes (48%), *FLT3* (30%), JAK genes (13%), and *FGFR1* (4%). Among these genes, *PTPN11* was the most frequently mutated (35%). Enrichment of these signaling mutations was more pronounced in AML-M0 than in non-M0 AML without core-binding factors or *KMT2A* rearrangements (Fig. 1B).

Notably, *RUNX1* and *ETV6* aberrations were frequently observed in immature pediatric leukemia subtypes, including AUL, T/myeloid mixed-phenotype acute leukemia (MPAL), and ETP-ALL (Fig. 1C), suggesting shared molecular features across immature acute leukemia phenotypes.

Additionally, we compared the genomic alterations in 23 pediatric AML-M0 patients with those in 34 adult AML-M0 patients (TCGA-LAML: *n* = 15; Beat AML: *n* = 19). Consistent with the pediatric cohort, *RUNX1* aberrations were the most prevalent genetic alteration in adult AML-M0 patients, occurring in 38% of cases (Supplementary Fig. S1). Biallelic *RUNX1* alterations were observed in 62% (8/13) of affected adult cases. These alterations included missense mutations within the DNA-binding Runt domain, nonsense mutations, and frameshift variants (Fig. 1D). In addition, *ETV6* loss-of-function aberrations (12%) occurred exclusively with *RUNX1* alterations, as observed in pediatric AML-M0. In contrast to pediatric AML-M0, adult cases had more frequent alterations in *TP53* (21%) and epigenetic and splicing regulators, such as *IDH2* (24%), *DNMT3A* (15%), and *ASXL1* (15%) (Supplementary Fig. S1).

### AML-M0 exhibits unique gene expression and DNA methylation profiles

**Fig. 2:**
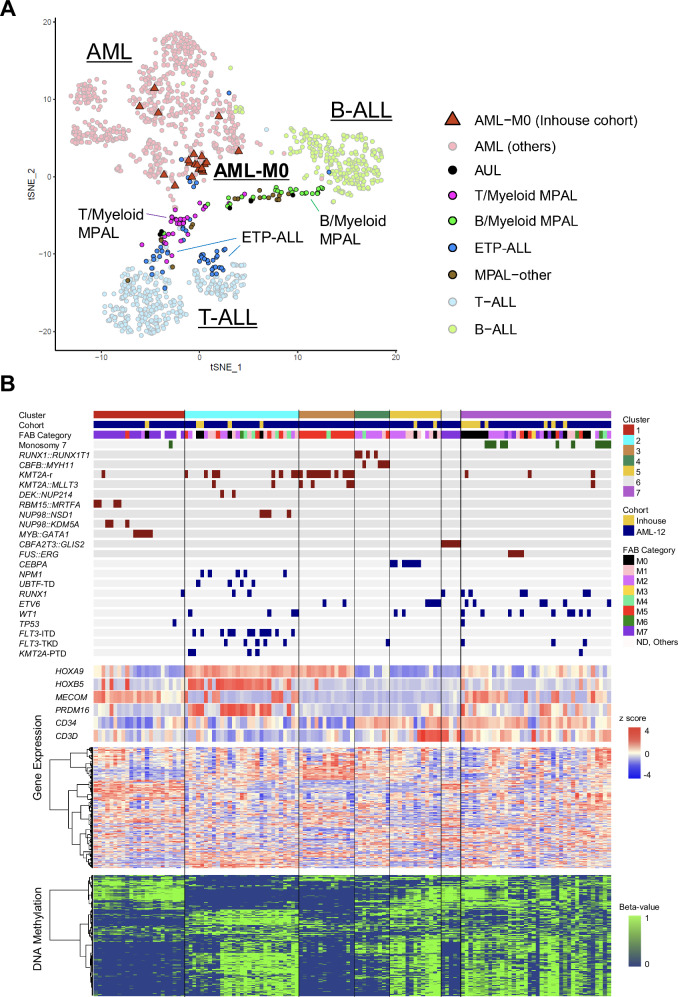
Comparisons of AML-M0 and other leukemic subtypes. **A** T-SNE projections of gene expression data from in-house samples and publicly available pediatric datasets (AML-M0, *n* = 19; non-M0 AML, *n* = 656; T-ALL, *n* = 388; B-ALL, *n* = 253; ALAL, *n* = 85). The 1000 most variable genes (based on absolute median deviation) were used with a perplexity of 100 to investigate global relationships. **B** Unsupervised hierarchical clustering of AML samples (*n* = 131) was performed using the 1000 most variably expressed genes and 1000 most variably methylated probes integrated through similarity network fusion.

To explore the molecular features of AML-M0, we analyzed its gene expression profile using RNA sequencing (RNA-seq) across various types of pediatric acute leukemia, including AML, T-ALL, B-ALL, and ALAL, which comprise AUL, T/myeloid MPAL, B/myeloid MPAL, and other MPAL subtypes. T-distributed stochastic neighbor embedding (t-SNE) analysis revealed three primary clusters corresponding to AML, T-ALL, and B-ALL, with ALAL samples distributed between these clusters (Fig. 2A). AML-M0 samples were localized within the AML cluster and were closely adjacent to ALAL, which was consistent with their immature phenotype and supported the current WHO-based classification in our cohort. Similarly, ETP-ALL clustered near ALAL within the T-ALL group, suggesting that leukemia with the immature phenotype represented a continuous molecular spectrum.

In the AML cohort, AML-M0 formed a transcriptionally distinct subgroup with a high pediatric leukemic stem cell (pLSC6) score [37] (Supplementary Fig. S2). Subsequently, we evaluated the DNA methylation profiles, which are known to reflect the molecular basis of the disease and are useful for AML subclassification [38]. Most AML-M0 samples were included in a coherent cluster and co-localized with those harboring monosomy 7, indicating shared epigenetic features (Supplementary Fig. S3).

To refine the classification and further clarify the relationship between AML-M0 and other AML subtypes, we integrated transcriptome and methylation data using similarity network fusion, followed by consensus clustering. AML-M0 samples primarily belonged to cluster 7, which was characterized by high *CD34* and *CD3D* expression, consistent with an immature phenotype (Fig. 2B). Notably, this cluster included samples with monosomy 7 or a *FUS::ERG* fusion gene, both of which are associated with a poor prognosis. AML-M0 cases in other clusters harbored subtype-specific alterations, including *CEBPA* mutations, *MYB::GATA1* fusions, and *FLT3* internal tandem duplications. Collectively, these findings highlight that AML-M0 is a heterogeneous subgroup but a major subset of AML-M0 cases, particularly those without defining alterations, shared similar gene expression signatures and DNA methylation profiles, reflecting an immature phenotype.

### AML-M0 is characterized by global DNA hypermethylation and downregulation of widespread biological processes

**Fig. 3:**
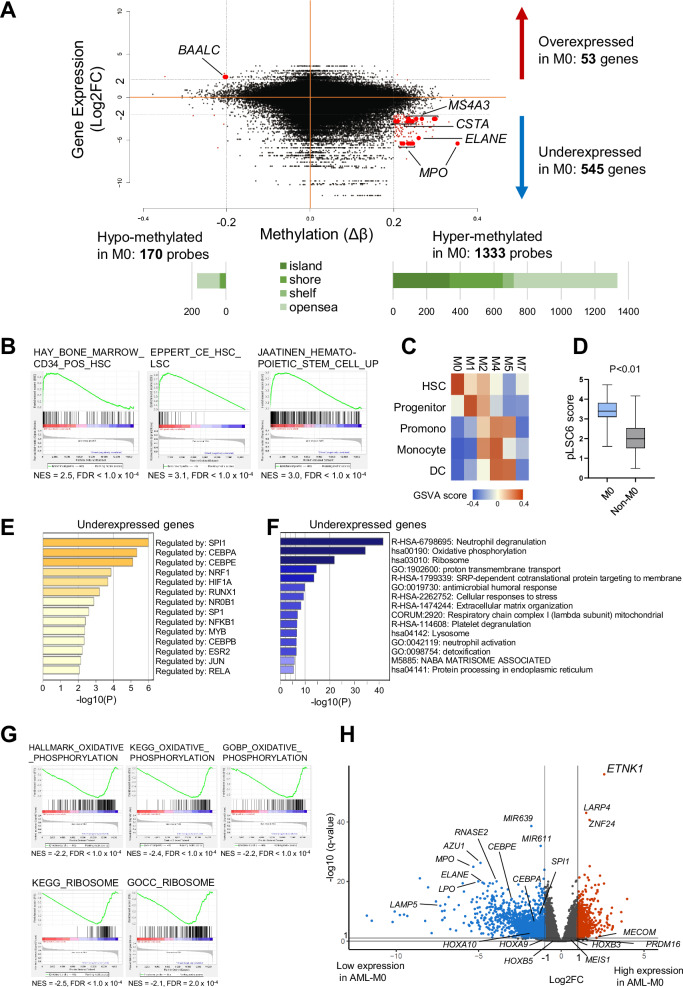
Gene expression and DNA methylation profiles of AML-M0. **A** Starburst plot illustrating gene expression and DNA methylation profiles in pediatric AML-M0 (gene expression, *n* = 19; DNA methylation, *n* = 20) compared to non-M0 pediatric patients with AML (gene expression, *n* = 389; DNA methylation, *n* = 116). Each dot represents a methylation probe, plotted according to the difference of methylation (Δβ) and corresponding gene expression (Log_2_FC). Probes with |Δβ|>0.2 and |Log_2_FC|>2 are highlighted as red dots. **B** Enrichment analysis of stemness-related signatures. **C** Enrichment analysis of myeloid developmental signatures according to the FAB classification. Enrichment scores were calculated for each individual sample using GSVA, and the median value for each signature in each group is displayed. **D** Comparison of pLSC6 scores between patients with AML-M0 and non-M0 AML. **E**, **F** Enrichment analysis was performed using the TRRUST and pathway/process databases in Metascape. A list of the 500 most significantly downregulated genes in AML-M0 cells was used. **G** Enrichment analysis using OxPhos and Ribosome-related gene sets was performed using GSEA. **H** Volcano plot illustrating differentially expressed genes between AML-M0 and non-M0 AML. Genes with a q-value < 10^–1^, and Log_2_FC > 1 or Log_2_FC < –1 are highlighted as red dots or blue dots, respectively.

To further elucidate the molecular characteristics of pediatric AML-M0, we performed a comparative analysis of the gene expression and DNA methylation profiles of pediatric non-M0 AML. Compared to non-M0 AML cells, AML-M0 cells display widespread DNA hypermethylation and reduced gene expression. A total of 1333 probes were significantly hypermethylated (q < 0.05, ΔB > 0.2), 25% of which were located on CpG islands. In addition, 545 genes were significantly downregulated, of which 77 were classified as underexpressed genes with hypermethylated probes (Fig. 3A). These “hypermethylated and under-expressed” genes included *MPO, CSTA, MS4A3*, and *ELANE*, all of which are closely related to myeloid differentiation (Supplementary Table S8). In contrast, overexpressed genes with hypomethylated probes included *BAALC*, a gene known to inhibit myeloid differentiation and associate with leukemogenesis, chemoresistance, and poor prognosis [39, 40]. The differentially methylated regions in these genes were not consistently located in the promoter regions (Supplementary Table S8). Therefore, the direct contribution of DNA methylation changes to the observed alterations in gene expression remains unclear.

GSEA and gene set variation analysis (GSVA) revealed that AML-M0 was enriched in stemness-associated signatures (Fig. 3B, C). These findings remained consistent when the analysis was restricted to cases from clusters 7 and 2 in Fig. 2B (Supplementary Fig. S4). Additionally, the pLSC6 score was significantly higher in AML-M0 patients than in non-M0 AML patients (Fig. 3D). Furthermore, enrichment analysis using the TRRUST database in Metascape revealed that the genes regulated by transcription factors associated with myeloid hematopoiesis (*SPI1, CEBPA, CEBPE*, and *RUNX1*) were significantly downregulated (Fig. 3E). These findings validate the stem-like properties of AML-M0 based on its gene expression profile and morphological and immunophenotypic features.

Widespread biological suppression is another hallmark of the disease. GSEA revealed the negative enrichment of 46 of the 50 hallmark gene sets in AML-M0 cells (Supplementary Fig. S5). Oxidative phosphorylation (OxPhos) and ribosome-related genes were markedly downregulated in the Metascape analysis (Fig. 3F), which was further validated using GSEA software (Fig. 3G). These findings were consistent even when the samples from Clusters 7 and 2 were analyzed independently (Supplementary Fig. S4). Notably, genes related to other metabolic processes, including glycolysis, were negatively enriched (Supplementary Fig. S5). Differentially expressed gene (DEG) analysis revealed upregulation of *ETNK1* (Fig. 3H), a gene involved in the conversion of ethanolamine to phosphoethanolamine, which inhibits mitochondrial respiration and the OxPhos system.

Similar trends were observed in adult AML datasets. Consistent with the pediatric cohort, adult AML-M0 cells were characterized by the significant downregulation of genes associated with myeloid differentiation and upregulation of stemness signatures, as demonstrated by DEG analysis, GSEA, GSVA, and the 17-gene leukemia stem cell (LSC17) scoring system [41] (Supplementary Fig. S6A–D). Adult AML-M0 cells also exhibited negative enrichment of numerous hallmark gene sets, notably the downregulation of genes associated with the OxPhos and glycolysis pathways. However, the ribosome-related genes were less affected (Supplementary Fig. S6E, F).

### Loss-of-function alterations of *RUNX1* are associated with poor prognosis in pediatric AML-M0

**Fig. 4:**
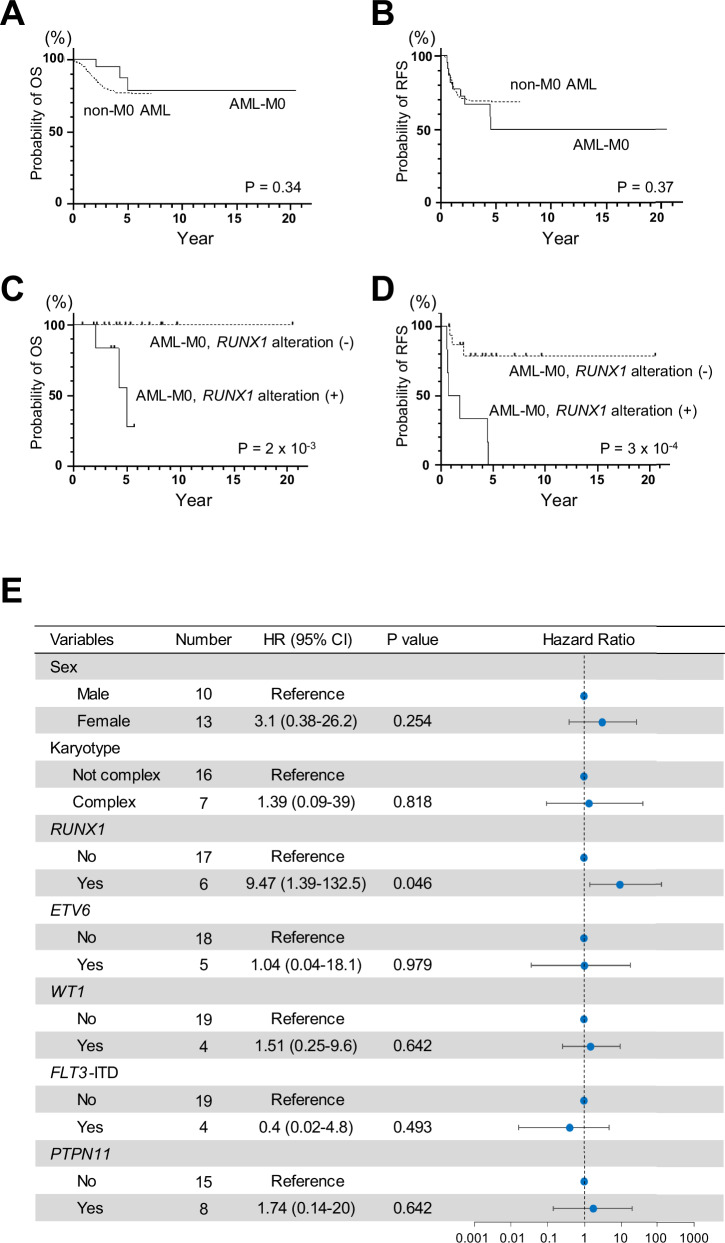
Survival analysis of AML-M0. Overall survival (**A**) and relapse-free survival (**B**) of in-house AML-M0 (*n* = 23) and non-M0 (*n* = 353) AML patients (JPLSG AML12). Overall survival (**C**) and relapse-free survival (**D**) of the in-house AML-M0 cohort comparing cases with (*n* = 6) and without (*n* = 17) *RUNX1* alterations. **E** Forest plot showing the results of multivariate Cox regression analysis of the effects of different parameters on relapse-free survival. Circles represent hazard ratios, and horizontal lines represent 95% confidence intervals.

Previous studies have reported that AML-M0 has a poor prognosis [4, 5]. To assess the clinical impact, we performed survival analysis using the AML-M0 and JPLSG AML-12 clinical trial cohorts. The 5-year overall survival (OS) was 78.9% for AML-M0 vs. 76.9% for non-M0 AML (*P* = 0.34; Fig. 4A), and the relapse-free survival (RFS) was 50.2% vs. 68.7% (*P* = 0.37; Fig. 4B). Notably, AML-M0 patients with *RUNX1* alterations had significantly lower OS (*P* = 2 × 10^–3^; Fig. 4C) and RFS (*P* = 3 × 10^–4^; Fig. 4D) than other AML-M0 patients. Cox regression analysis identified *RUNX1* alterations as an independent risk factor for relapse in AML-M0 (HR = 6.96; 95% CI, 1.31–62.3), whereas other frequent alterations, such as *ETV6*, *WT1*, and *PTPN11*, were not significantly associated with survival (Fig. 4E).

The clinical characteristics did not differ between the *RUNX1*-altered and *RUNX1*-wildtype patients (Supplementary Table S1). All six AML-M0 patients with *RUNX1* alterations were treated using the JPLSG AML-05 or AML-12 protocols. Two patients underwent hematopoietic stem cell transplantation as part of their initial treatment, and subsequently relapsed. Three patients were treated with conventional chemotherapy only, two of whom relapsed within one year and one within five years. One patient harboring the t(3;3) (p25;q26.2) translocation experienced primary induction failure.

To explore the molecular mechanisms underlying the poor prognosis of *RUNX1*-altered AML-M0 patients, we compared the gene expression and DNA methylation profiles of *RUNX1*-altered and *RUNX1*-wildtype patients (Supplementary Fig. S7A). The “hypermethylated and underexpressed” genes included *CSTA* and *MS4A3*, both of which are associated with myeloid differentiation (Supplementary Fig. S7B). On the other hand, “hypomethylated and overexpressed” genes included *BAALC, DNTT*, and *BLNK*, which are typically expressed in immature hematopoietic progenitors. GSEA also showed the enrichment of stemness-related gene sets; however, there was no clear difference in the expression of OxPhos- or ribosome-related genes (Supplementary Fig. S7C). These results suggested that *RUNX1* loss may promote an undifferentiated phenotype in AML-M0 cells.

We performed survival analysis in adult AML cohorts to determine whether a similar trend was observed in adult patients. Although *RUNX1* alterations were associated with a trend toward poorer prognosis, the differences were not statistically significant (Supplementary Fig. S8).

### *RUNX1* depletion downregulates ribosome- and OxPhos-related pathways and reduces sensitivity to chemotherapy

**Fig. 5:**
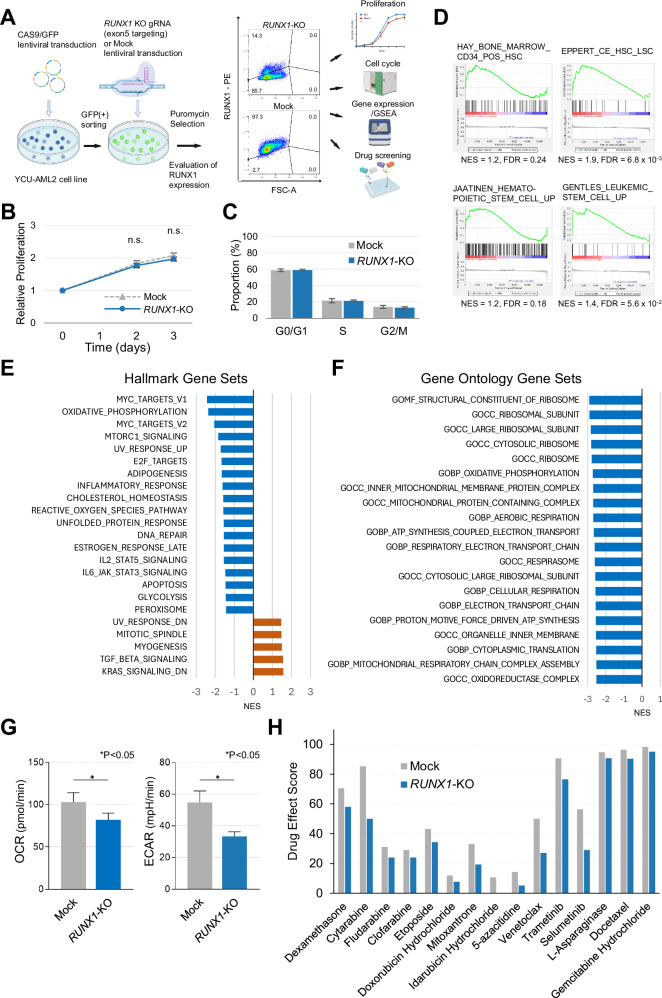
*RUNX1* knockout in AML-M0 cell line “YCU-AML2.”. **A** *RUNX1* knockout YCU-AML2 cells were established via lentiviral transduction with Cas9 and sgRNAs targeting exon 5 of the *RUNX1* gene. Three independent knockout clones were compared with mock-transduced control cells. RUNX1 protein expression levels were analyzed using intracellular flow cytometry. A schematic representation was created using https://biorender.com. **B** Cell proliferation assay comparing *RUNX1*-knockout (blue) and mock control (gray) cells. **C** Cell cycle assay of *RUNX1*-knockout (blue) and mock control (gray) cells. **D** Enrichment analysis of stem cell-related signatures using GSEA. **E** Enrichment analysis of hallmark gene sets calculated using GSEA. Gene sets with q-value < 0.1 are shown. **F** Enrichment analysis of Gene Ontology (GO) gene sets calculated using GSEA. The top 20 negatively enriched gene sets in *RUNX1*-knockout cells are shown. **G** Seahorse extracellular flux analysis of YCU-AML2 cells comparing *RUNX1*-knockout (blue) and mock control (gray). Oxygen consumption rate (OCR; left) and extracellular acidification rate (ECAR; right) were measured under basal conditions using the Seahorse XF Real-Time ATP Rate Assay Kit. **H** Sensitivity to representative drugs is presented as a drug effect score by comparing *RUNX1*-knockout cells (blue) and mock-transfected control cells (gray).

To validate the pathogenic role of *RUNX1* alterations, we performed in vitro experiments using the recently established pediatric AML-M0 cell line, YCU-AML2 [35], which lacks *RUNX1* alterations. The CRISPR/Cas9 system targeting the Runt domain of *RUNX1* achieved more than 85% knockout efficiency with multiple frameshift alterations (Fig. 5A; Supplementary Fig. S9A and Supplementary Table S9).

Short-term cell proliferation and cell cycle assays showed no significant differences between *RUNX1*-knockout and mock control cells (Fig. 5B, C). In contrast, clonogenic assays demonstrated a reduced colony-forming capacity in *RUNX1*-knockout cells (Supplementary Fig. S9B). As stemness signatures were upregulated in *RUNX1*-altered AML-M0 cells in clinical samples, the upregulation of leukemic stem cell signatures was observed in these in vitro experiments. (Fig. 5D). GSEA using hallmark gene sets revealed downregulation of genes involved in multiple biological pathways, including the OxPhos and ROS pathways in *RUNX1*-knocked-out cells (Fig. 5E). Additionally, GSEA using Gene Ontology gene sets showed a marked downregulation of ribosome-related and OxPhos-related gene sets in *RUNX1*-knocked-out cells (Fig. 5F).

To functionally assess the metabolic changes indicated by our transcriptomic analyses, we performed a Seahorse extracellular flux assay. Both the OCR and ECAR were reduced in *RUNX1*-knockout cells compared to controls, supporting the coordinated attenuation of oxidative phosphorylation and glycolytic activity (Fig.5G and Supplementary Fig. S9C).

Finally, we performed a drug-screening test using *RUNX1*-knockout YCU-AML2 cells. Drug sensitivity testing revealed that *RUNX1* disruption induced resistance to multiple drugs, including key drugs for AML treatment such as cytarabine and anthracyclines (Fig. 5H and Supplementary Fig. S9D). Resistance to the MEK inhibitors Trametinib and Selumetinib was also observed. In contrast, L-Asparaginase, Docetaxel, and Gemcitabine remain effective despite not being used in standard AML treatment regimens.

These findings suggest that *RUNX1* loss-of-function increases stemness and impairs OxPhos and ribosome function, potentially leading to reduced ROS and DNA damage, which could contribute to chemoresistance in AML-M0 cells.

## Discussion

This study presents the first comprehensive molecular characterization of pediatric AML-M0, a rare and high-risk leukemia with a poorly understood biology. By integrating multi-omics profiling and functional assays, we described a distinct stem cell-like profile characterized by global DNA hypermethylation and broad transcriptional repression, and identified *RUNX1* alterations as key drivers of chemoresistance and poor prognosis, offering novel avenues for targeted therapies.

Downregulation of OxPhos and ribosome-related genes was observed in AML-M0 cells, reflecting a stem cell-like molecular state supported by the enrichment of hematopoietic stem cell (HSC)-associated gene expression signatures. This phenotype aligns with normal hematopoiesis, in which DNA methylation is the highest in HSCs, OxPhos is suppressed to limit ROS production, and translation is downregulated to maintain stemness [42, 43].

Frequent *RUNX1* and *ETV6* alterations were observed in pediatric AML-M0, consistent with previous pediatric and adult reports [8, 11]. Both transcription factors are essential for myeloid differentiation, and their disruption impairs hematopoiesis [44–46]. This likely contributes to leukemogenesis, whereas the co-occurring signaling mutations drive proliferation. Importantly, we identified *RUNX1* alterations as a novel adverse prognostic factor in pediatric AML-M0 patients, consistent with its association with poor outcomes in pediatric and adult AML [47–50]. Our in vitro experiments suggest that *RUNX1* depletion suppresses genes associated with ribosomal and ROS-related pathways, thereby conferring chemoresistance. This is consistent with mouse models, in which Runx1 directly regulates ribosome biogenesis; its loss reduces ribosome production in HSCs and progenitor cells [51], leading to resistance against genotoxic and endogenous stress. This mechanism is consistent with previous findings that leukemic stem cells evade chemotherapy by suppressing ROS despite high mitochondrial metabolism [52].

Previous studies on CCG-2891 and CCG-2961 conducted by the Children’s Oncology Group reported inferior outcomes for AML-M0 [4]. Conversely, our cohort did not show a poor prognosis. Most patients in our study were treated according to the JPLSG AML-05 or AML-12 protocols [15, 16], which resulted in improved clinical outcomes. This suggests that advances in treatment strategies may mitigate the historically poor prognosis. Furthermore, previous studies may have included patients with AUL who responded less effectively to AML-type chemotherapy, potentially contributing to inferior outcomes observed in historical cohorts.

The distinct molecular characteristics of AML-M0 cells suggest several potential therapeutic strategies. The hypermethylated state may indicate potential sensitivity to hypomethylating agents such as 5-azacytidine. Frequent signaling mutations indicate the feasibility of targeted approaches, including MEK and FLT3 inhibitors, tailored to mutational profiles. Suppressed ribosomal function may confer sensitivity to translation inhibitors, such as pegcrisantaspase or homoharringtonine, particularly in *RUNX1*-mutated cases [53, 54]. BCL2 inhibitors have shown synergistic effects with these agents; notably, a phase 1 study of pegcrisantaspase plus venetoclax achieved complete remission in all four patients with *RUNX1*-mutated AML [53]. Furthermore, the observed downregulation of both OxPhos and glycolysis suggests the potential efficacy of metabolism-targeted therapies, including venetoclax and 5-azacytidine [55].

Our study had several limitations. First, the number of available cases was limited due to the rarity of pediatric AML-M0, which restricted the statistical power of some analyses and precluded validation in an independent external cohort. Moreover, AML-M0 in this study was defined based on morphology and immunophenotype, which is not fully equivalent to the current WHO category of “acute myeloid leukemia with minimal differentiation,” and this difference should be considered when interpreting the results.

Second, the functional experiments should be regarded as exploratory and preliminary because they were performed using a single AML-M0 cell line. YCU-AML2 is the only available AML-M0 cell line used in this study and harbors a *KMT2A::MLLT3* rearrangement. This genetic background rarely co-occurs with *RUNX1* mutations in clinical samples, and therefore differs substantially from the molecular context observed in our patient cohort. Under short-term culture conditions, no substantial differences in cell growth were observed between *RUNX1*-knockout and control cells. In contrast, *RUNX1*-knockout resulted in a reduced proliferative capacity in colony-forming assays, consistent with its essential role as a core hematopoietic transcription factor required for leukemic cell maintenance [56]. Consistent with this observation, seahorse extracellular flux analysis demonstrated reduced oxidative phosphorylation and glycolytic activity in *RUNX1*-knockout cells, indicating coordinated metabolic attenuation. Such growth suppression may reflect a dose-dependent effect of perturbing key hematopoietic transcription factors and may be analogous to findings reported for *GATA2*, where partial loss delays leukemia initiation, but is associated with a more aggressive disease phenotype in vivo [57]. Although a similar mechanism could operate following *RUNX1* perturbation, definitive conclusions could not be drawn from this study.

Finally, the drug sensitivity assay was limited in scope and experimental scale, precluding definitive conclusions regarding therapeutic vulnerabilities. Collectively, these factors limit the generalizability of the findings and underscore the need for further validation using additional experimental models, including other cell lines, primary patient samples, and in vivo approaches.

Despite these limitations, our study provides important insights into the molecular pathogenesis of pediatric AML-M0 and potential therapeutic strategies. To the best of our knowledge, this is the largest comprehensive molecular characterization of pediatric AML-M0 patients to date.

In summary, our comprehensive analysis identified that AML-M0 cells exhibit shared genetic and epigenetic features associated with an immature stem cell-like phenotype beyond their morphological and immunophenotypic characteristics. We highlight the prognostic impact of *RUNX1* alterations and their potential role in chemoresistance, providing a basis for novel therapeutic approaches to improve treatment strategies in this high-risk subgroup.

## Supplementary information

Supplementary Figures

Supplementary Table S1

Supplementary Table S2

Supplementary Table S3

Supplementary Table S4

Supplementary Table S5

Supplementary Table S6

Supplementary Table S7

Supplementary Table S8

Supplementary Table S9

Supplementary Methods

## Data Availability

The datasets generated and/or analyzed in the current study are available in the Japanese Genotype-Phenotype Archive (JGA), hosted by the DNA Data Bank of Japan (DDBJ) under accession code JGAS000867.

## Code availability

All software and bioinformatics tools used in this manuscript are publicly available.
