# Supplementary material for: Multi-omics analysis of pediatric minimally differentiated acute myeloid leukemia reveals RUNX1-driven stemness and chemoresistance
Source: Leukemia. 2026 Apr 29;40(7):1427–38. doi: 10.1038/s41375-026-02967-6 (PMC13322964; doi:10.1038/s41375-026-02967-6)
Supplement: Supplementary file 1 — Supplementary Figures [file 41375_2026_2967_MOESM1_ESM.pdf]

Supplementary Figure S1

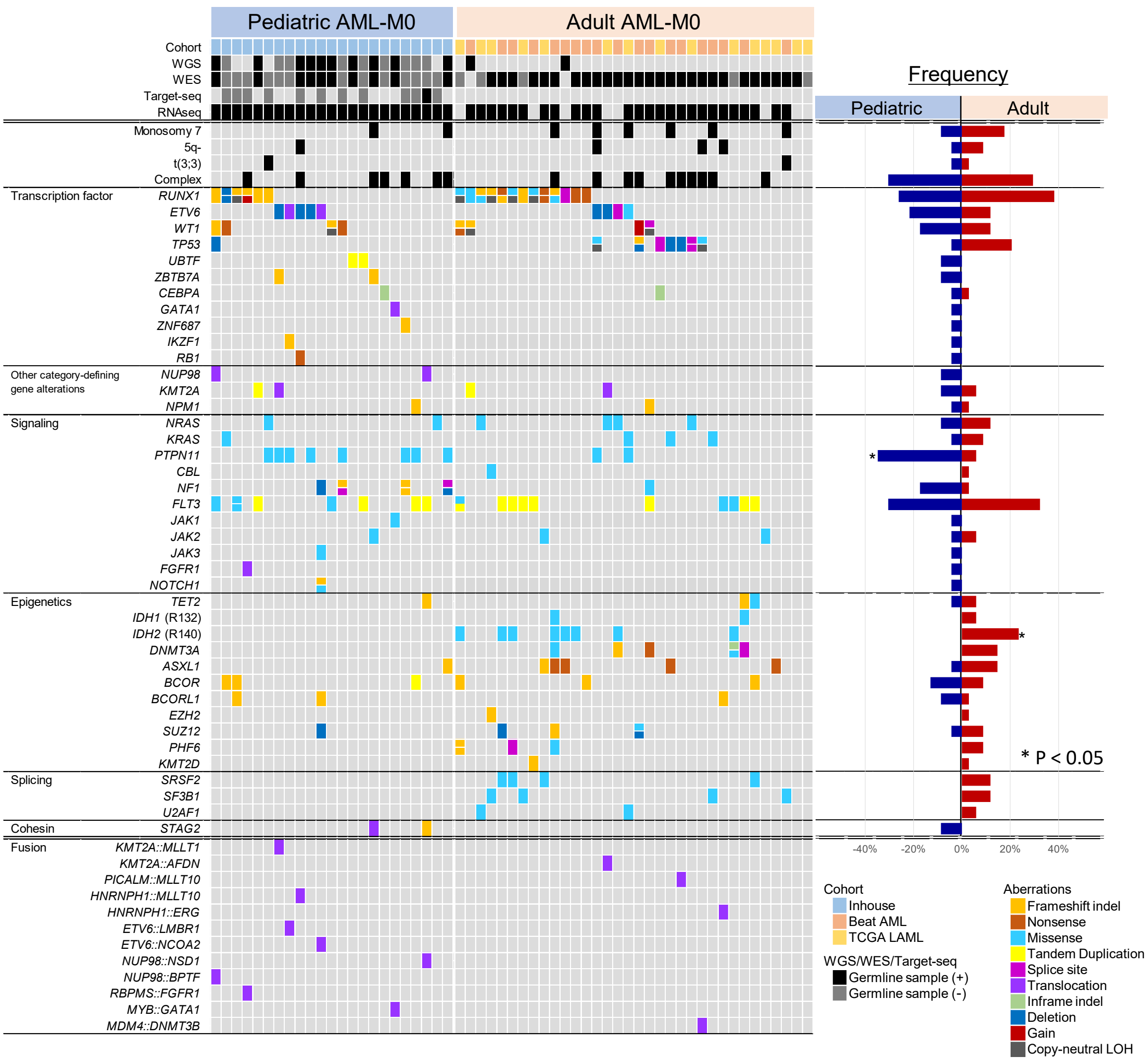

Supplementary Figure S1. Comparative landscape of genomic alterations in pediatric and adult AML-M0

The landscape of genomic alterations in 23 pediatric and 34 adult AML-M0 patients, the frequencies of which are shown on the right-hand side.

Supplementary Figure S2

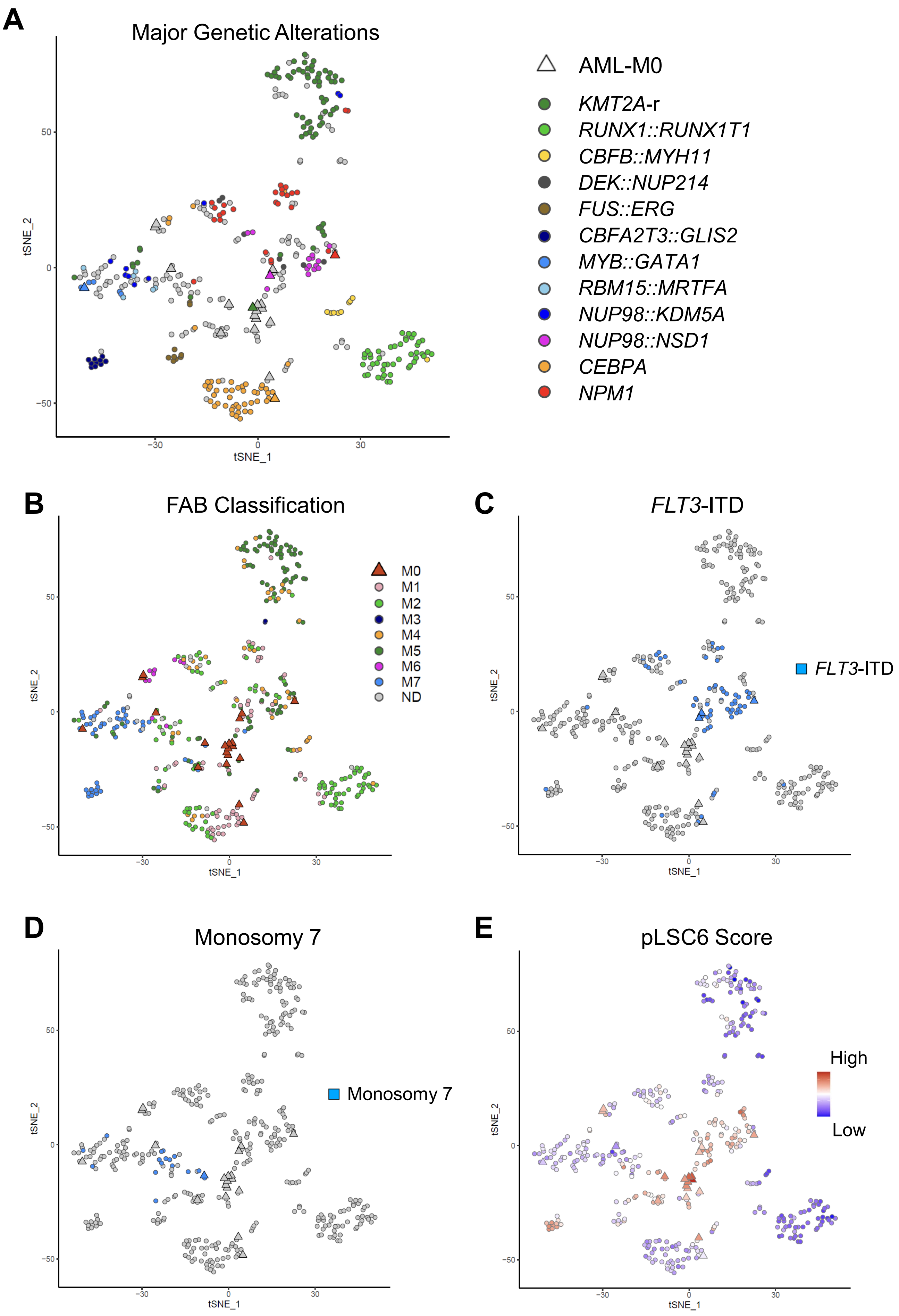

Supplementary Figure S2. t-SNE projection of pediatric AML using gene expression data
A-E. T-SNE projection of gene expression data from in-house samples and publicly available pediatric AML datasets. The top 1,000 most variably expressed genes (based on the absolute median deviation) were used. Triangles represent the AML-M0 samples. The colors of each dot denote (A) molecular categories, (B) FAB Classification, (C) samples harboring *FLT3*-ITD, (D) samples with monosomy 7, and (E) the pLSC6 score of each sample.

Supplementary Figure S3

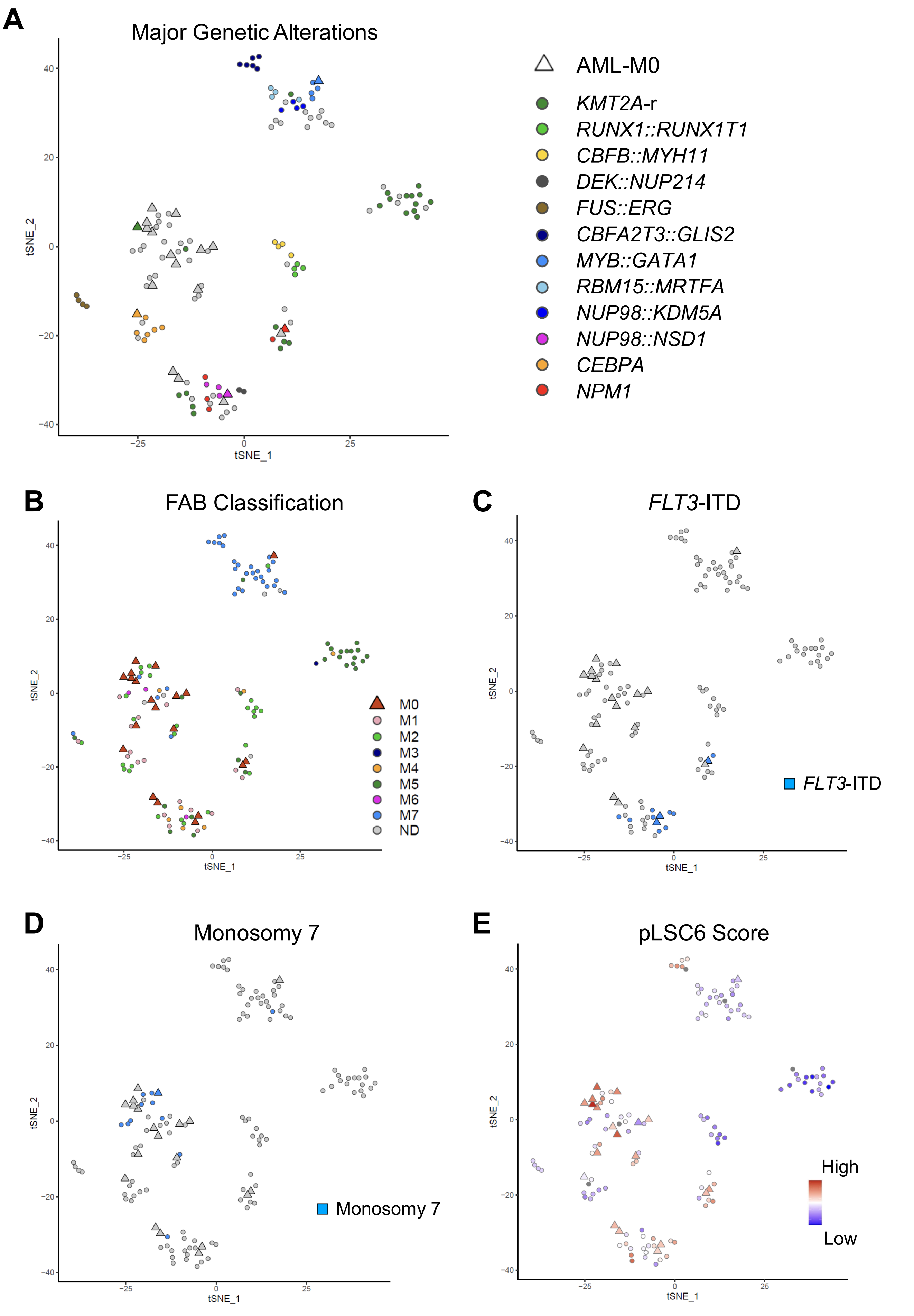

Supplementary Figure S3. t-SNE projection of pediatric AML using DNA methylation data

A-E. T-SNE projection of DNA methylation data from in-house samples and publicly available pediatric AML datasets. The top 1,000 variably methylated probes (based on absolute median deviation) were used. Triangles represent the AML-M0 samples. The colors of each dot denote (A) molecular categories, (B) FAB Classification, (C) samples harboring *FLT3*-ITD, (D) samples with monosomy 7, and (E) the pLSC6 score of each sample.

Supplementary Figure S4

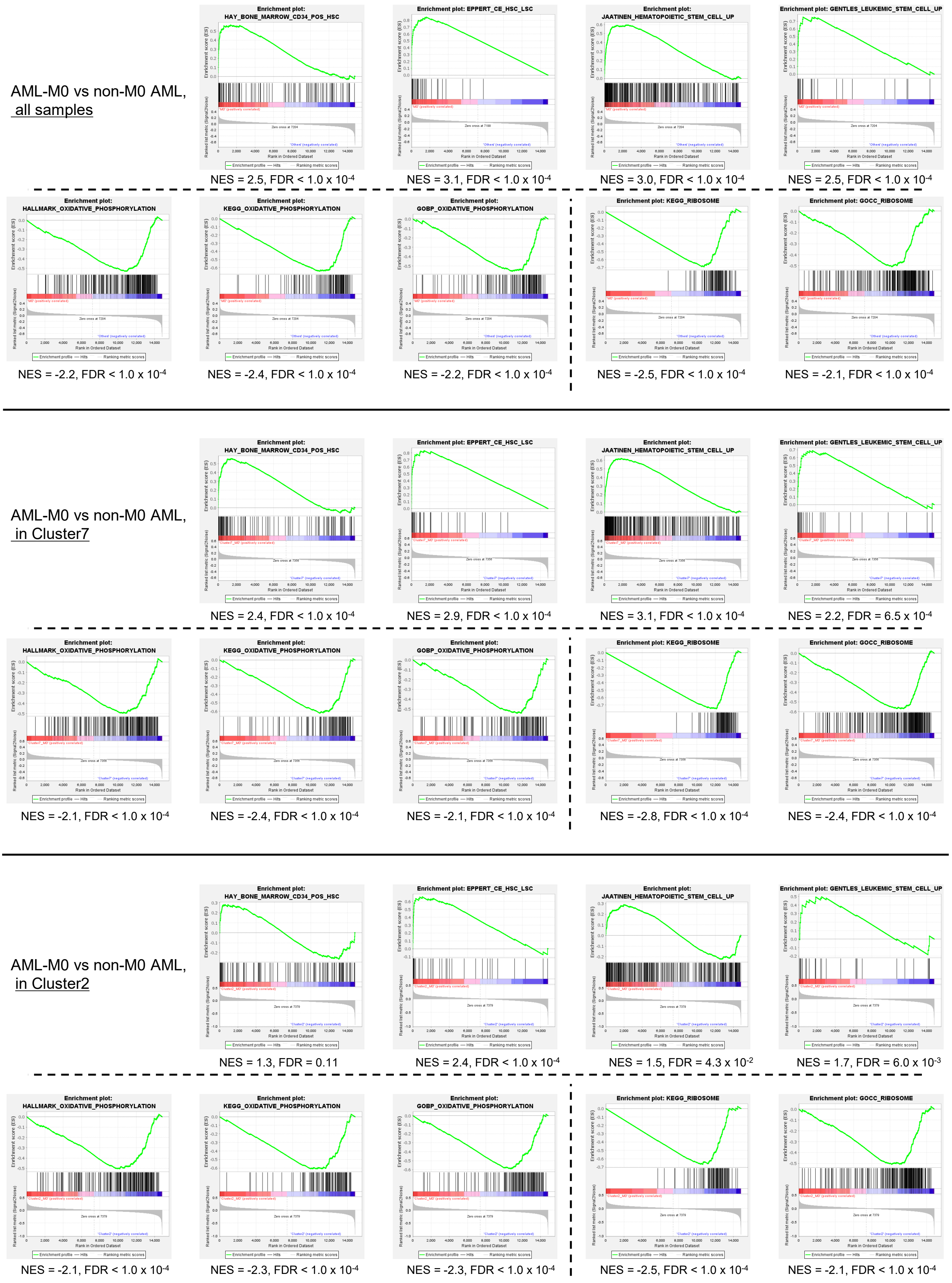

Supplementary Figure S4. Gene Set Enrichment Analysis comparing pediatric AML-M0 and non-M0 AML

Enrichment plots, normalized enrichment scores, and false discovery rates are shown as results of GSEA using gene sets associated with hematopoietic stem cells, leukemic stem cells, oxidative phosphorylation, and ribosomes. Top: Results for all the samples. Middle: Results using samples from Cluster 7 of the integrated analysis (Fig. 2B). Bottom: Results using samples from Cluster 2 of the integrated analysis (Fig. 2B).

Supplementary Figure S5

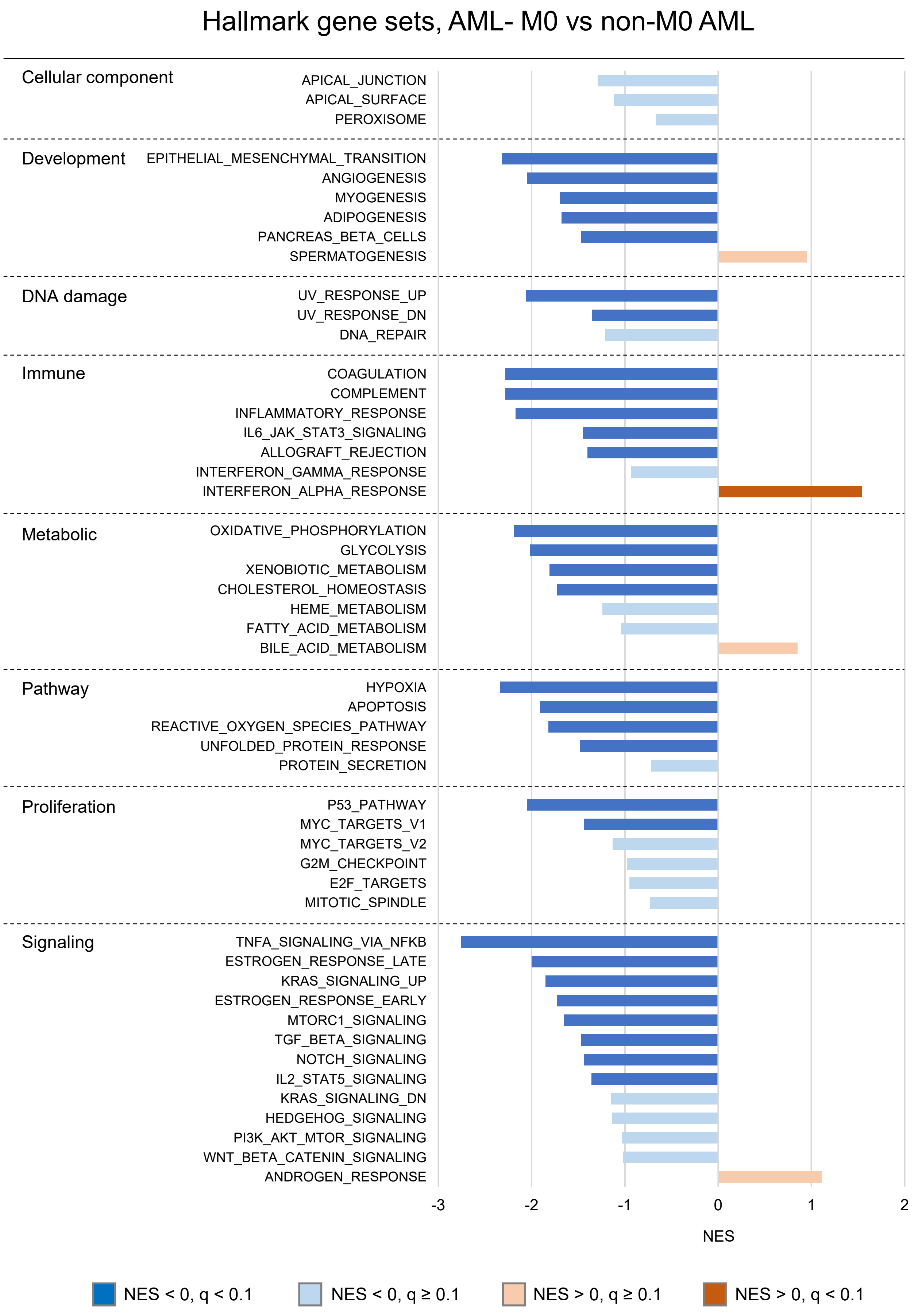

Supplementary Figure S5. Gene Set Enrichment Analysis of hallmark gene sets comparing pediatric AML-M0 and non-M0 AML

The bar chart shows normalized enrichment scores (NES) of GSEA using all hallmark gene sets comparing AML-M0 and non-M0 AML. The colors are based on NES and false discovery rate (q-value).

Supplementary Figure S6

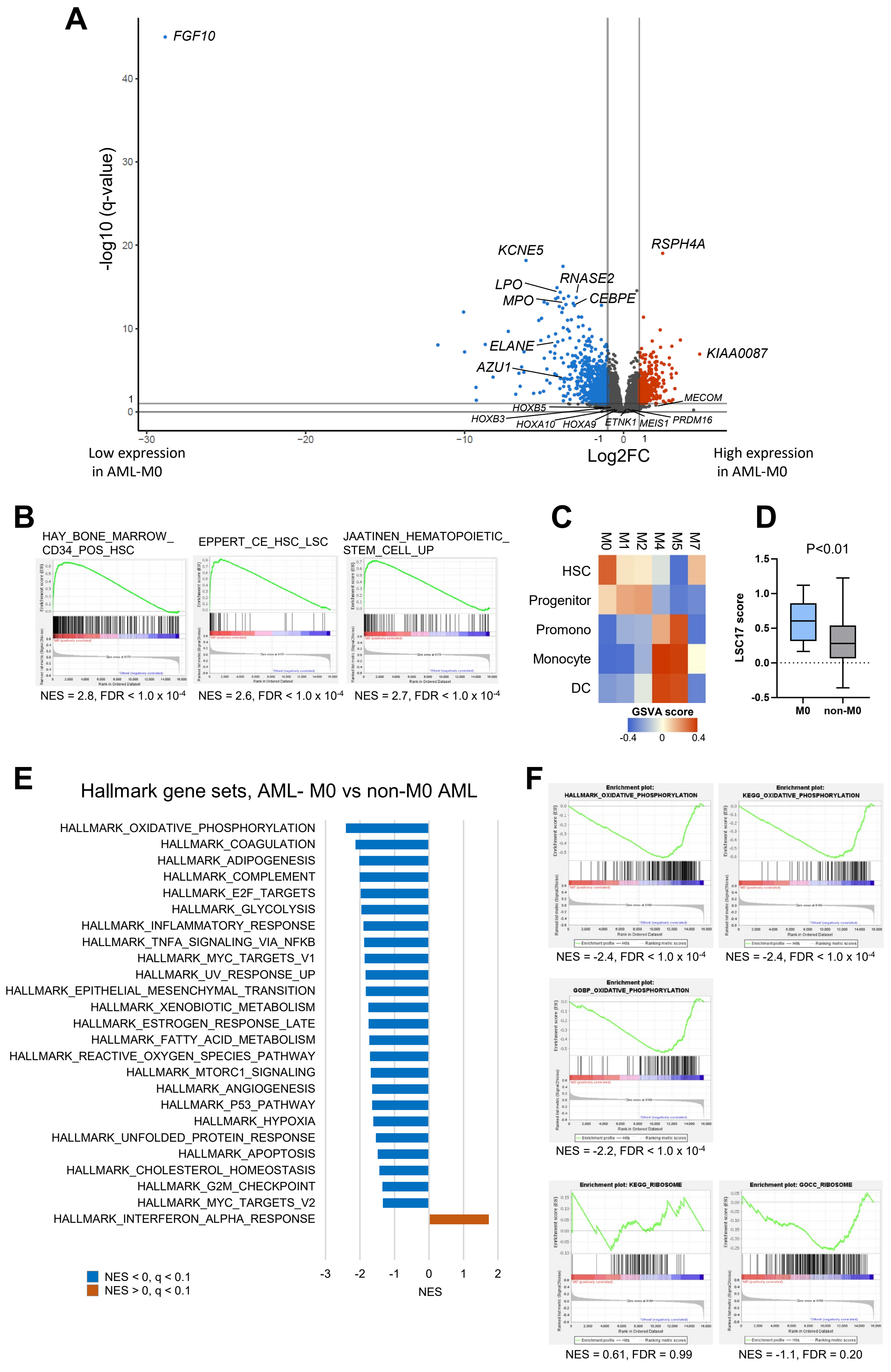

Supplementary Figure S6. Comparative analysis of AML-M0 and non-M0 AML in adult cohorts

A. Differentially expressed genes were visualized using volcano plots. Genes with a q-value $<10^{-1}$, and $Log_2FC > 1$ or $Log_2FC < -1$ are highlighted as red dots or blue dots, respectively. **B.** Enrichment analysis of stemness-related signatures **C.** Enrichment analysis of myeloid developmental signatures according to the FAB classification. Enrichment scores were calculated for each individual sample using GSVA, and the median value for each signature in each group is displayed. **D.** Comparison of the LSC17 scores between AML-M0 and non-M0 AML patients. **E.** Enrichment analysis of Gene Ontology gene sets calculated using GSEA. Gene sets with q-values <0.1 are displayed. **F.** Enrichment analysis using OxPhos and Ribosome-related gene sets calculated by GSEA.

Supplementary Figure S7

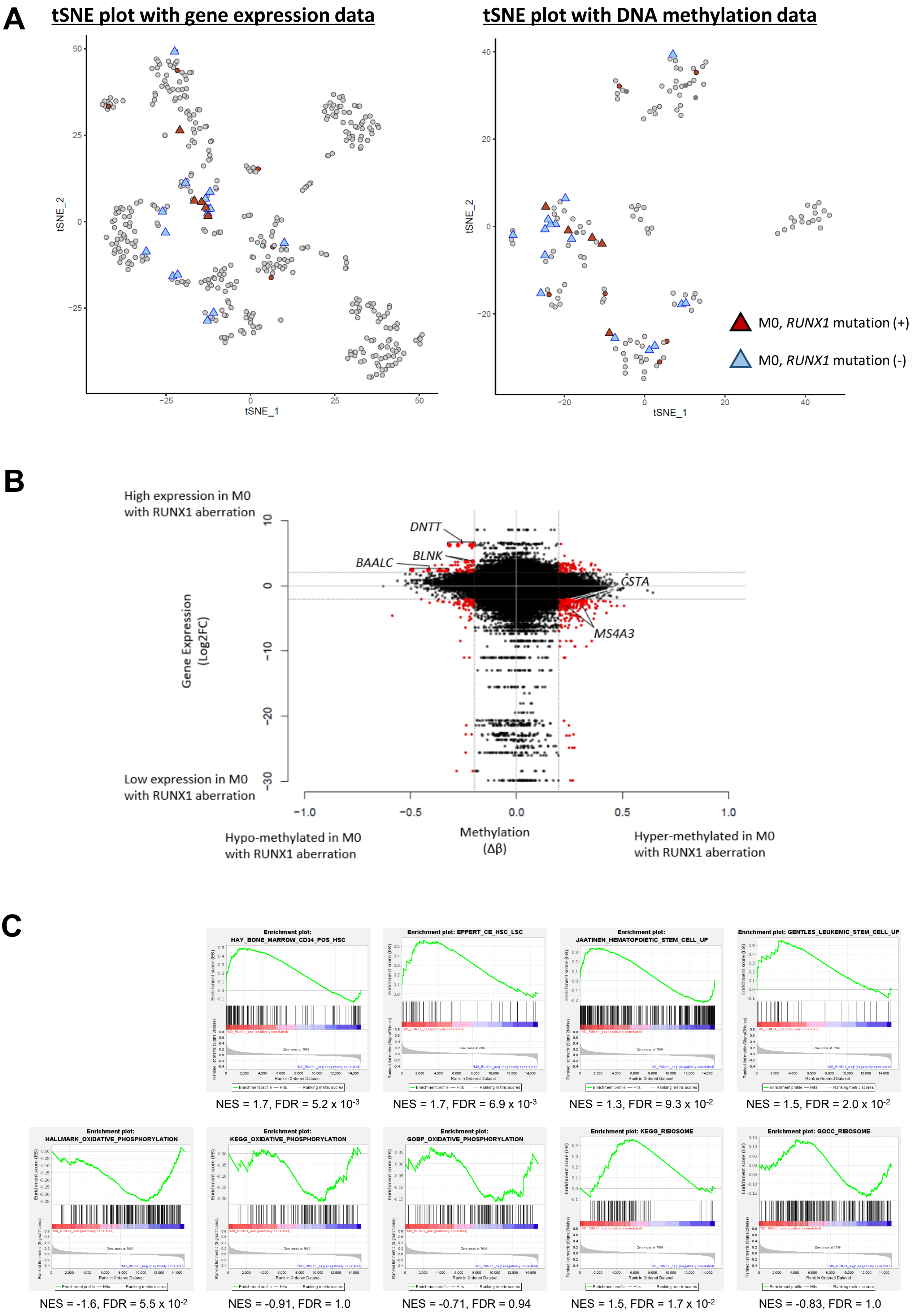

Supplementary Figure S7. Comparative analysis of pediatric AML-M0 with and without *RUNX1* alterations

A. t-SNE projection of gene expression data (left) or DNA methylation data (right) showing AML-M0 samples (triangles) and those with *RUNX1* alterations (red). **B.** Starburst plot depicting gene expression and DNA methylation data comparing AML-M0 cells with and without *RUNX1* aberration. Each dot represents a methylation probe, plotted according to difference of methylation ($\Delta\beta$) and corresponding gene expression (Log_2FC). Probes with $|\Delta\beta| > 0.2$ and $|Log_2FC| > 2$ are highlighted as red dots. **C.** Enrichment analysis of the signatures associated with stemness, OxPhos, and ribosomal function.

Supplementary Figure S8

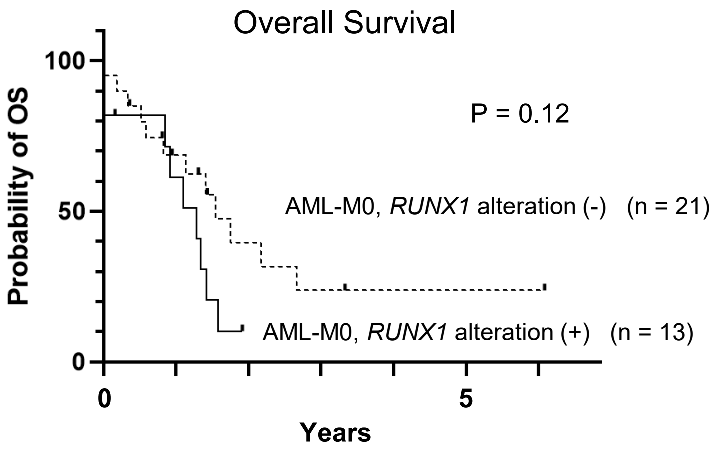

Supplementary Figure S8. Survival analysis of adult AML-M0 cohort
Overall survival of adult AML-M0 cohort comparing cases with (n=13) and without (n=21) *RUNX1* alterations.

Supplementary Figure S9

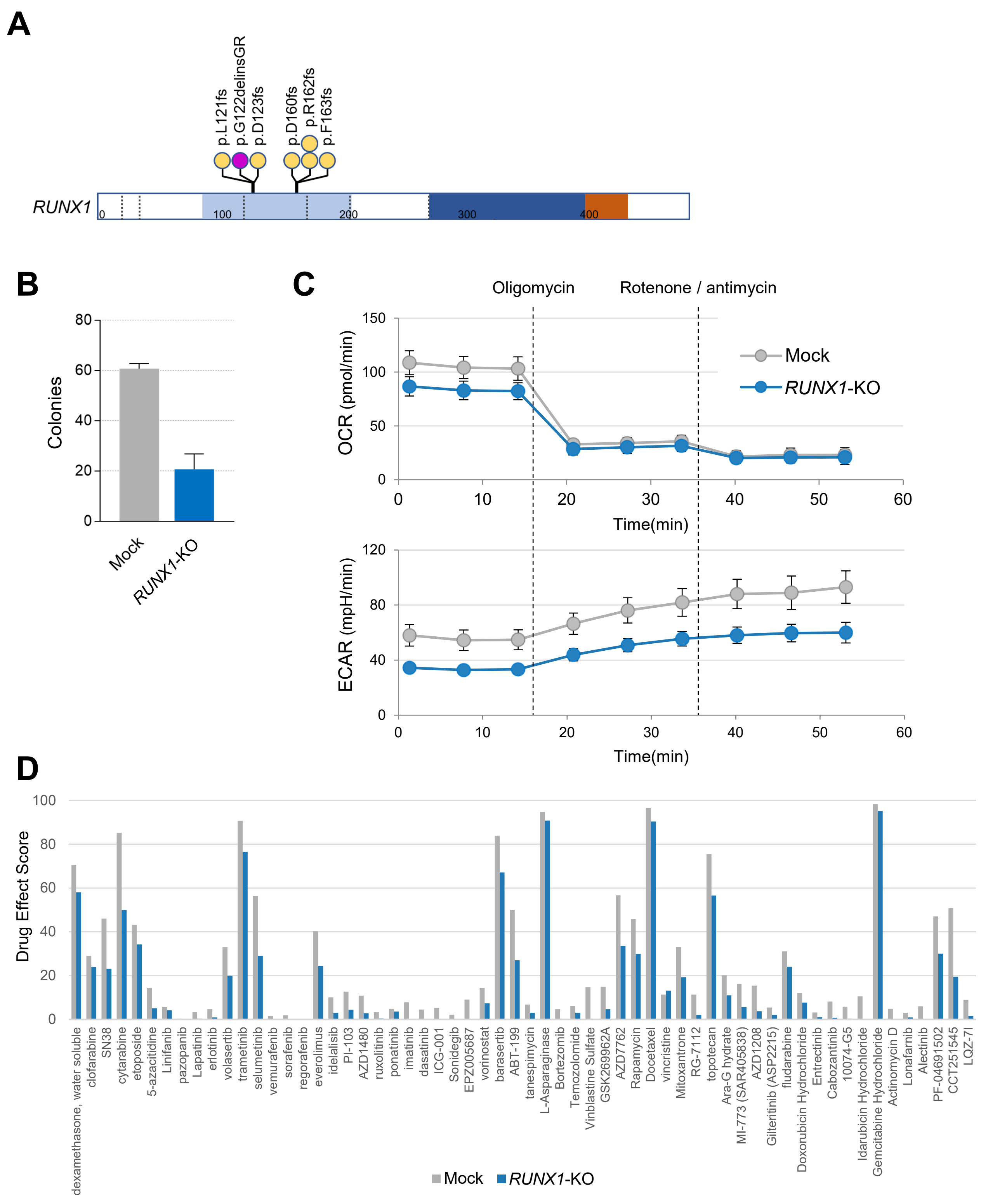

Supplementary Figure S9. In vitro functional analysis of RUNX1 in AML-M0

A. Summary of *RUNX1* alterations detected in *RUNX1*-knocked-out cells. **B.** The number of colonies formed in clonogenic assay comparing *RUNX1*-knocked-out cells (blue) and mock-transfected control cells (gray). **C.** Oxygen consumption rate (OCR; upper) and extracellular acidification rate (ECAR; lower) measured in seahorse extracellular flux assay, comparing *RUNX1*-knocked-out cells (blue) and mock-transfected control cells (gray). **D.** Drug sensitivity analysis for all tested drugs is presented as a drug effect score comparing *RUNX1*-knocked-out cells (blue) and mock-transfected control cells (gray).
