# Supplementary material for: Multi-omics analysis of pediatric minimally differentiated acute myeloid leukemia reveals RUNX1-driven stemness and chemoresistance
Source: Leukemia. 2026 Apr 29;40(7):1427–38. doi: 10.1038/s41375-026-02967-6 (PMC13322964; doi:10.1038/s41375-026-02967-6)
Supplement: Supplementary file 11 — Supplementary Methods [file 41375_2026_2967_MOESM11_ESM.docx]

**Supplementary Methods**

**Patients and materials**

A total of 31 pediatric AML-M0 cases, diagnosed as “M0” in the FAB classification and/or “AML with minimal differentiation” in the WHO classification at each hospital, were initially selected for analysis. The diagnosis was subsequently confirmed through a central review of the JCCG and evaluation of flow cytometry data. Eight cases were excluded: six were reclassified as non-M0 AML subtypes according to the FAB classification, one was diagnosed with AUL upon central review, and one lacked sufficient data to confirm the diagnosis. Consequently, 23 pediatric AML-M0 patients were included in the analysis. Morphologically, all patients met the criteria for “M0” in FAB classification. According to the diagnostic criteria of the WHO classification [3], blast cells must express at least two myeloid-associated markers (CD13, CD117, and/or CD33) and be negative for lymphoid markers. Based on these criteria, seven of 23 AML-M0 cases were finally classified in the “AML with minimal differentiation” category, and sixteen were in the “AML with defining genetic abnormalities” category under the WHO classification. Before sample collection, written informed consent was obtained from patients or their guardians. This study was conducted in accordance with the Declaration of Helsinki and approved by the ethics committees of the JCCG and Kyoto University. Seven and six patients were enrolled in the AML-05 and AML-12 clinical trials of the Japanese Pediatric Leukemia and Lymphoma Study Group (JPLSG, now part of the subcommittee on hematological malignancies of the JCCG), respectively [15, 16]. Clinical and sample information, as well as sequencing details, are provided in Supplementary Table S1. A detailed analysis of case no. 9 was reported previously [14].

Additionally, we analyzed the sequencing data from pediatric acute leukemia and adult AML cohorts [13, 15-24]. Japanese AML data were derived from the JPLSG AML-12 and AML05 cohorts and deposited in the JGA under accession codes JGAS000867 and in the European Genome-Phenome Archive (EGA), which is hosted by the European Bioinformatics Institute (EBI) under accession number EGAS00001003701. Japanese B-ALL data were derived from the Japan Association Childhood Leukemia Study Group (JACLS) ALL-02 or Tokyo Children’s Cancer Study Group (TCCSG) L04-16 clinical trials and deposited in the JGA under the accession number PRJDB8942. Japanese T-ALL data were also derived from the JACLS and TCCSG cohorts and deposited in the JGA under accession number JGAS00000000090. The data generated by the TARGET initiative (AML, B-ALL, T-ALL, and ALAL) are available under accession number phs000218, managed by the National Cancer Institute. Adult AML data were obtained from the GDC Data Portal under accession numbers phs000178 (TCGA-LAML) and phs001657 (BEATAML1.0-COHORT). Target capture sequencing (Target-seq) data from the JPLSG AML-12 cohort (N=357, including six AML-M0 cases) were analyzed to compare the genomic profiles of AML-M0 with those of non-M0 AML subtypes (Figs. 1A–C). Additionally, a published list of alterations in TARGET-ALAL (AUL and T/myeloid MPAL) and Japanese T-ALL (ETP and non-ETP phenotypes) was used to compare the frequencies of alterations (Fig. 1D). RNA-seq data from pediatric AML cohorts, including JPLSG AML-12 (N=254), JPLSG AML-05 (N=139), and TARGET-AML (N=271), as well as those from Japanese T-ALL (N=123) and B-ALL (N=116) cohorts and TARGET-ALL (N=86 for ALAL, N=265 for T-ALL, and N=137 for B-ALL), were utilized for the t-SNE projection of gene expression data (Fig. 2A). RNA-seq and DNA methylation data from the JPLSG AML-12 cohort (N=117) were analyzed using integrated unsupervised clustering (Figs. 2B and 3A). In addition, RNA-seq data from the JPLSG AML-12 and JPLSG AML-05 cohorts were used to compare the gene expression profiles of AML-M0 and non-M0 AML subtypes (Figs. 3B–H). The FAB classifications of all cases included in JPLSG AML-12 and AML-05 cohorts were strictly based on a central review by the JCCG.

**DNA sequencing**

Tumor and germline DNA were extracted from the bone marrow or peripheral blood mononuclear cells of patients using a QIAamp DNA Mini Kit (QIAGEN) according to the manufacturer’s instructions. WES, low-depth WGS, and high-depth WGS were performed in 23, 15, and 4 cases, respectively. For library preparation of WES and low-depth WGS, 50 ng of DNA was used with the Lotus™ DNA Library Prep Kit for NGS (Integrated DNA Technologies, IDT). The xGen™ Hybridization and Wash Kit and xGen Exome Hyb Panel v2 (IDT) were used for exon capture in WES library preparation. Sequencing was performed on a DNBSEQ-G400RS (MGI Tech) in 150 base-pair paired-end mode, following the manufacturer’s instructions. For library preparation for high-depth WGS, the TruSeq DNA PCR-Free Library Prep Kit (Illumina) and Illumina DNA PCR-Free Prep Tagmentation (Illumina) were used, followed by sequencing using a NovaSeq 6000 in 150 base-pair paired-end mode.

The target depths for the tumor and normal samples in WES, low-depth WGS, and high-depth WGS were 150x, 100x, 45x, 30x, 100x, and 30x, respectively. The actual median (range) depths of the tumor and normal samples in WES, low-depth WGS, and high-depth WGS were 141x (103–252x), 116x (94–131x), 47x (37–57x), 31x (19–39x), 126x (122–144x), and 30x (29–31x), respectively.

The sequencing reads were aligned to the human reference genome (GRCh37/hg19) using the Burrows-Wheeler Aligner (v.0.7.8) with default parameter settings. Single nucleotide variants (SNVs) and short insertion/deletions (indels) were called using the Genomon pipeline v.2.6 (https://github.com/Genomon-Project) with the following parameters: (1) Mapping quality score ≥20; (2) Base quality score ≥15; (3) Both tumor and normal (if available) depths ≥8; (4) Number of variant reads in tumors ≥4; (5) Strand ratio not equal to 0 or 1. The candidate variants were further filtered by excluding the following: (i) Variant allele frequency (VAF) in tumor samples <0.05; (ii) EBCall P value >10^-4^; (iii) known variants listed in SNP databases (1000 Genome Project, Exome Sequencing Project 6500, Human Genetic Variation Database, Exome Aggregation Consortium, and integrative Japanese Genome Variation Database); (iv) variants out of coding regions or splice sites; (v) synonymous and ambiguous SNVs. If a paired germline sample was available, variants with (vi) VAF ≥0.1 in the germline sample, and (vi) Fisher’s exact test P value >10^−1^ were excluded. The excluded variants were re-included when they (a) had five or more entries in the Catalogue of Somatic Mutations in Cancer (COSMIC) Database version 90; (b) were Pathogenic or Likely Pathogenic variants in the ClinVar database; and/or (c) were frameshift, nonsense, or splice-site variants in genes with reported recurrent loss-of-function aberrations^11^. If a paired germline sample was unavailable, only the variants that met criteria (a), (b), and/or (c) were included. Finally, mapping errors were excluded by visual inspection using the Integrative Genomics Viewer (IGV) browser.

For Target-seq data, alignment of sequencing reads and calling of SNVs and indels were performed as described for WES/WGS. Germline data were not available. The variants were filtered by excluding the following: (i) VAF < 0.02, (ii) EBCall P-value > 10^-4^; (iii) known variants listed in SNP databases (detailed above), (iv) variants outside the coding regions or splice sites, and (v) synonymous and ambiguous SNVs. Candidate variants were further filtered by removing variants with a VAF of 0.4–0.6 in copy-neutral regions, except variants meeting the following criteria: (a) had five or more entries in the COSMIC Database version 90; (b) were Pathogenic or Likely Pathogenic variants in ClinVar database; and/or (c) were frameshift, nonsense, or splice-site variants in genes with reported recurrent loss-of-function aberrations. Finally, the mapping errors were excluded by visual inspection using an IGV browser.

**Detection of copy number alterations and structural variations**

Copy number alterations (CNAs) were analyzed using sequencing data, with WES and Target-seq processed through our in-house pipeline, CNACS (https://github.com/papaemmelab/toil_cnacs), and WGS analyzed using the CNVkit [25]. Structural variations were detected using Genomon pipeline (v.2.6) and filtered by excluding (i) Fisher’s exact test P-value >10^-1^ when paired germline samples were available, (ii) variations outside the coding regions or splice sites, and (iii) variations observed in unpaired normal samples. Additionally, the mapping errors were manually reviewed and excluded by visual inspection using an IGV browser.

**RNA sequencing**

Total RNA was extracted using QIAzol Lysis Reagent and the miRNeasy Mini Kit (QIAGEN) following the manufacturer’s protocol. Library preparation was performed with 100 ng RNA using the NEBNext Poly(A) mRNA Magnetic Isolation Module and NEBNext Ultra II RNA Library Prep Kit for Illumina (New England Biolabs). For Case #5, in which the RNA quality was compromised (RIN integrity number <4.0), the NEBNext rRNA Depletion Kit (New England Biolabs) was used instead of the Poly(A) mRNA isolation module. Sequencing was performed on a DNBSEQ-G400RS (MGI Tech) in 150 base-pair paired-end mode, following the manufacturer’s instructions.

Sequencing reads were aligned to GRCh37/hg19 and counted for each gene using the Genomon pipeline (version 2.6). Fusion transcripts were detected using the Genomon pipeline (version 2.6), and previously reported leukemia-associated fusions were extracted.

**Gene expression analysis**

Three cases (#5, #16, and #22) were excluded from gene expression analysis due to their low tumor content (<50%). Genes with Transcripts Per Kilobase Million (TPM) <1.0, in 100% of the samples or a mean TPM <1.0, were classified as low-expression genes and excluded. Gene expression levels were normalized, transformed using the R package DESeq2 [26], and subjected to subsequent analyses. Differential expression analysis was performed using the DESeq2 package. Gene set enrichment analysis (GSEA) was performed using GSEA software [27] (v4.3.2) and the Molecular Signatures Database [28, 29] (v2023.2). A pre-ranked GSEA was performed using the Metascape software [30] (v3.5.20240901) (http://metascape.org/). Single-sample-level gene set enrichment was calculated using the R package GSVA [31] with default parameters. Multiple subtypes of pediatric leukemia were analyzed in both the Japanese leukemia and TARGET cohorts. A comparative analysis of pediatric AML-M0 and non-M0 AML was performed using only Japanese AML cohorts, as their FAB subtypes were confirmed by a central review of the JCCG/JPLSG.

**DNA Methylation analysis**

Genomic DNA (250 ng) was bisulfite converted using an EZ DNA Methylation Kit (Zymo Research). The converted DNA was then amplified, enzymatically fragmented, purified, and hybridized to the Infinium MethylationEPIC v1.0 BeadChip (Illumina) according to the manufacturer’s protocols. Imaging was performed using an iScan system (Illumina). Data filtering, imputation, and normalization were conducted, and the β-values were calculated using the R package ChAMP [32] (v2.20.1) with default parameters. Differentially methylated probes (DMPs) were identified using the champ.DMP function in the ChAMP package. Probe annotations for the EPIC arrays were sourced from the Infinium MethylationEPIC v1.0 B5 Manifest File (https://jp.support.illumina.com/downloads/infinium-methylationepic-v1-0-product-files.html).

**Dual-omics integrative clustering**

For integrative unsupervised clustering, the top 1 000 variably expressed genes and top 1 000 most variably methylated probes were selected based on their median absolute deviations. The rlog and M-value matrices were z-score-standardized, and the Euclidean distances between the samples were calculated. These distance matrices were converted into affinity matrices and fused using the similarity network fusion algorithm implemented in the R package SNFtool [33] with the following parameters: alpha = 0.5, K = 10, and t = 20. The resulting integrated matrix was subjected to consensus clustering using the R package ConsensusClusterPlus [34] with the following parameters: reps = 1000, pItem = 0.8, clusterAlg = “hc,” innerLinkage = “ward.D2”, finalLinkage = “ward.D2”, distance = “euclidean.”

**Cells and cell culture**

YCU-AML2 [35] and derivative cell lines were cultured at a humidified incubator (37 °C/5% CO2) in RPMI 1640 medium supplemented with 10% fetal calf serum (FCS) and 1% penicillin/streptomycin.

***RUNX1* knock out using CRISPR-Cas9**

*RUNX1*-knockout cells were generated through sequential transduction of Cas9 and guide RNA (gRNA) targeting *RUNX1*. Stable expression of Cas9 in YCU-AML2 cells (YCU-AML2-Cas9) was achieved by transduction with a lentiviral vector, pKLV2-EF1a-Cas9GFP-W (Addgene plasmid #200100) in the presence of 5 µg/mL polybrene. Subsequently, the GFP-positive cells were sorted using an SH800 cell sorter (SONY). Two sgRNA sequences targeting exon 5 of *RUNX1* (Supplementary Table S2) were cloned into pKLV-U6gRNA(Bbsl)-PGKpuro2ABFP (Addgene plasmid #50946) and co-transduced into the YCU-AML2-Cas9 cells with 5 µg/mL of polybrene. Mock control cells were generated using original #50946 vector. Following transduction, the cells were subjected to puromycin selection (1 µg/mL) for 7 days. Cells expressing high GFP were sorted to enrich the *RUNX1* knocked-out population.

**Flow cytometric analysis of RUNX1 expression**

For each sample, 8×10^5^ cells were used to evaluate RUNX1 expression by flow cytometry, and dead cells were stained with eBioscience Fixable Viability Dye eFluor 780 (Thermo Fisher Scientific, 65-0865-14) to detect viable cells, following the manufacturer’s protocol. Then the cells were fixed and permeabilized using eBioscience Foxp3 / Transcription Factor Staining Buffer Set (Thermo Fisher Scientific, 00-5523-00) and stained with either AML1 (D33G6) XP® Rabbit mAb (PE Conjugate) (#15002) (1:500) (Cell Signaling Technology) or Rabbit (DA1E) mAb IgH XP® Isotype Control (PE conjugate) (1:500) (Cell Signaling Technology), according to the manufacturer’s instructions. Samples were resuspended in 500 µL phosphate-buffered saline (PBS) and measured using FACSLyric™ (Becton Dickinson). The flow cytometry results were analyzed using FlowJo v10.8 Software (BD Biosciences).

**Cell proliferation assay**

Cell proliferation was analyzed using Cell Counting Kit-8 (Dojindo Laboratories) according to the manufacturer’s instructions. Suspended cells were cultured in triplicate at a density of 2.0×10^5^ cells/mL with a total volume of 100 µL for each well in flat-bottomed 96-well plates (20,000 cells per well). The cells were cultured with medium containing 10% FCS in a humidified incubator (at 37 °C, 5% CO_2_) for 2, 3, or 4 days. After a 4-h incubation following the addition of 10 µL of WST-8 reagent to each well, the sample absorbance was measured at 450 nm using an iMark Microplate Reader (Bio-Rad). Absorbance values were normalized by dividing them with the absorbance on day 1, and the relative values were used to evaluate cell proliferation. All experiments were performed in triplicate as technical replicates, and the figures show the mean values with standard deviations.

**Cell cycle assay**

Cell cycle assays were performed using a Cell Cycle Assay Kit (Red) (Tokyo Chemical Industry Co., Ltd.) according to the manufacturer’s protocol. Briefly, 1.0×10^4^ cells were treated with 7-AAD (7-Amino-Actinomycin D) to stain intracellular DNA. DMSO-treated cells were used as controls. Stained cells were analyzed using a FACSLyric (Becton Dickinson). The flow cytometry results were analyzed using FlowJo v10.8 Software (BD Biosciences). The Dean–Jett–Fox algorithm was used to estimate the proportion of cells in the G0/G1, S, and G2/M phases of the cell cycle. All experiments were performed in triplicate as technical replicates, and the figures show the mean values with standard deviations.

**Colony formation assay**

Colony formation assays were performed using YCU-AML2 cells and MethoCult H4434 Classic (STEMCELL Technologies) according to the manufacturer’s instructions. All experiments were performed in triplicate as technical replicates, and the figures show the mean values with standard deviations.

**Seahorse extracellular flux assay**

The real-time ATP assay was performed using a Seahorse XF Real-Time ATP Rate Assay Kit (Agilent Technologies, 103592-100) according to the manufacturer’s instructions on a Seahorse XFe96 Analyzer. YCU-AML2 cells were suspended in XF RPMI medium (pH 7.4; Agilent Technologies, 103576-100) supplemented with 10 mM glucose (Agilent Technologies, 103577-100), 1 mM pyruvate (Agilent Technologies, 103578-100) and 2 mM L-glutamine (Agilent Technologies, 103579-100). 1.5 × 10e5 cells were added into each well of Seahorse XFe96/XF Pro Cell Culture Microplates (Agilent Technologies, 103794-100) coated with Cell-Tak Cell and Tissue Adhesive (Corning, 354240) and incubated for one hour at 37 °C in a non-CO2 incubator prior to the assay. Oxygen consumption rate (OCR) and extracellular acidification rate (ECAR) were measured at baseline and following the sequential injection of oligomycin (1.5 µM) and rotenone/antimycin A (0.5 µM) at the indicated time points.

**High-throughput drug sensitivity screening**

High-throughput drug sensitivity screening was performed as described in the previous report [36]. Briefly, cells were seeded at 1 × 10e4 viable cells/10 µL onto a 384-well plate that was preloaded with 10 µl of culture medium containing 1 of 60 drugs at 4 serially diluted concentrations (× 1, × 1/5, × 1/25, × 1/125). The drugs and their final concentrations are listed in Supplementary Table S3. After a 4-day incubation period, cell viability was assessed using the CellTiter-Glo luminescent assay with a GloMax plate reader (Promega). To compare drug sensitivities between samples the drug effect score (DES) was utilized as follows: DES = [(100 − % survival at 1/125 dilution) * ln(125) + (100 − % survival at 1/25 dilution) * ln(25) + (100 − % survival at 1/5 dilution) * ln(5) + (100 − % survival at no dilution)] / [ln(125) + ln(25) + ln(5) + 1]. A DES of 100 indicated that the vector killed all cells at every tested concentration, whereas a DES of 0 indicated that the drug had no effect. As a modification of the previously reported assay, which evaluated 81 compounds [36], we excluded drugs that exhibited minimal inter-sample variability and optimized the panel to allow testing on a single 384-well plate. Due to limited resource availability, this drug sensitivity screening was performed once as an exploratory experiment.

**Statistical analysis**

Statistical analyses were performed using the GraphPad Prism software (version 9.5.1). Unless otherwise specified, the association between categorical variables was tested using Fisher’s exact test. The Mann–Whitney U test was used to compare quantitative variables. All statistical tests were two-sided. In the survival analysis, the survival curve was estimated using the Kaplan–Meier method, and the log-rank test was used to compare outcomes. Multivariate analysis was performed using Cox regression analysis.

**Code availability**

All software and bioinformatics tools used in this manuscript are publicly available.
